# Membrane curvature induces cardiolipin sorting

**DOI:** 10.1038/s42003-019-0471-x

**Published:** 2019-06-20

**Authors:** Elena Beltrán-Heredia, Feng-Ching Tsai, Samuel Salinas-Almaguer, Francisco J. Cao, Patricia Bassereau, Francisco Monroy

**Affiliations:** 1 Departamento de Estructura de la Materia, Física Térmica y Electrónica, Universidad Complutense de Madrid, Plaza de Ciencias, 1, 28040 Madrid, Spain; 2 Departamento de Química Física, Universidad Complutense de Madrid, Avda. Complutense, s/n, 28040 Madrid, Spain; 3 Laboratoire Physico Chimie Curie, Institut Curie, PSL Research University, CNRS UMR168, 75005 Paris, France; 4 Instituto Madrileño de Estudios Avanzados en Nanociencia, IMDEA Nanociencia, Calle Faraday, 9, 28049 Madrid, Spain; 5 Sorbonne Université, UPMC Univ Paris 06, 75005 Paris, France; 6 Unit of Translational Biophysics, Instituto de Investigación Sanitaria Hospital Doce de Octubre (imas12), Avda. de Córdoba, s/n, 28041 Madrid, Spain; 7 Institute for Quantitative Biosciences-QB3, University of California at Berkeley, Berkeley, CA 94720 USA

**Keywords:** Membrane biophysics, Research data

## Abstract

Cardiolipin is a cone-shaped lipid predominantly localized in curved membrane sites of bacteria and in the mitochondrial cristae. This specific localization has been argued to be geometry-driven, since the CL’s conical shape relaxes curvature frustration. Although previous evidence suggests a coupling between CL concentration and membrane shape in vivo, no precise experimental data are available for curvature-based CL sorting in vitro. Here, we test this hypothesis in experiments that isolate the effects of membrane curvature in lipid-bilayer nanotubes. CL sorting is observed with increasing tube curvature, reaching a maximum at optimal CL concentrations, a fact compatible with self-associative clustering. Observations are compatible with a model of membrane elasticity including van der Waals entropy, from which a negative intrinsic curvature of −1.1 nm^−1^ is predicted for CL. The results contribute to understanding the physicochemical interplay between membrane curvature and composition, providing key insights into mitochondrial and bacterial membrane organization and dynamics.

## Abstract (web summary)

Elena Beltrán-Heredia, Feng-Ching Tsai et al. examine the role of cardiolipin in membrane curvature, finding evidence compatible with a model based on membrane elasticity and van der Waals entropy. These results promote understanding of the interplay between membrane curvature and composition.

## Introduction

Cardiolipin (CL) is a negatively charged lipid found predominantly in the inner mitochondrial membrane of eukaryotic cells^1^ and in the plasma membrane of some bacteria^2^. The CL molecule is composed of two phosphatidic acids linked together by a short glycerol bridge, which results in a conical shape with a smaller cross-sectional area in the polar head relative to the hydrophobic tails. Previous estimates suggest that the intrinsic curvature of CL should be negative with an absolute value in the nanometer range^2–4^, making this molecule prone to localize in highly concave regions of the lipid membrane. In bacterial cells, CL-enriched domains have been observed to localize at the poles and division sites^4–6^. Furthermore, CL participates in the binding of some peripheral proteins placed on these highly curved regions^7^. Although these facts are suggestive for a possible role of CL in bacterial cell shaping^4,5^, however, no evidence of its essential contribution to cell division has been raised from experiments. Indeed, neither significant defects in cell division are detected in CL-deficient mutants of *E. coli*^8^, nor CL-specific domains are observed in *B. subtilis*^9^. In eukaryotic cells, CL is related to the maintenance of tubular-like invaginations, e.g. the mitochondrial *cristae*, which stabilize protein complexes necessary for respiration and oxidative phosphorylation^10,11^ and for the synthesis of ATP^11,12^. The proposed mechanism to form CL-enriched cristae domains is customary assumed to be geometry-driven^13^; arguably, a coupling between CL curvature and local composition would optimize the bending elasticity of the mitochondrial membrane.

However, due to their small size relative to proteins, lipids alone are generally not expected to be curvature sensitive except for in very particular conditions^14,15^. Some studies have shown that CL-containing bilayers are prone to create folds and adopt highly curved structures^16^, which are favored by the presence of divalent cations or low pH^17,18^. Experimental observations with membrane models have shown how cristae-like invaginations are induced by flowing protons to GUVs containing CL^19,20^. The coupling between CL concentration and membrane shape is also supported by the evidence of the accumulation of CL in *E. coli* minicell membranes^21^. At a molecular level, recent simulations of the bilayer structure by molecular dynamics (MD) reveal that the CL molecule, with a high intrinsic curvature due to its four acyl chains, is expected to concentrate at curved regions of the membrane. Differently, phosphatidylethanolamine (PE), another cone-shaped lipid with only two chains, seems to have much a weaker propensity to localize in these curved regions^13^. A quantitative estimation of the intrinsic curvature and sorting energy of CL molecules is biologically relevant as far it could contribute to understand whether this functional lipid can be sorted by curvature, and its protein-specificity could influence the spatial distribution of certain proteins. Curvature-driven sorting has been extensively explored in vitro for typical lipid systems^14,15,22,23^, as well as for membrane embedded proteins^24–26^. Although lipid sorting could be mediated by a coupling between membrane composition and curvature^27–29^, experimental and theoretical works show how mixing entropy tends to homogenize the lipid distribution^14,24,30^. Previous works noticed that it is very unlikely that lipids, unassisted by interactions, may be enriched in curved regions simply based on their shape alone^14,24^. Instead, lipid-lipid or lipid-protein-interactions appear to play an essential role in getting the membrane susceptible to curvature-driven lipid sorting. Indeed, it has been shown that lipid sorting only occurs if the system is close to a phase-separation point, when lipid-lipid binary interactions become dominant and the separation process is amplified upon clustering with proteins^14,24^. These analyses have been made with different lipid species commonly found in membrane cells, however, CL has not been so far examined in this context. Furthermore, the practical use of non-specific fluorescent probes, like the mitochondrial dye NAO, has been evidenced to be unlikely to generalize conclusions about CL-localization in bacteria^8,9^, which calls for a revision of the specific role of CL in bacterial cell division.

**Fig. 1:**
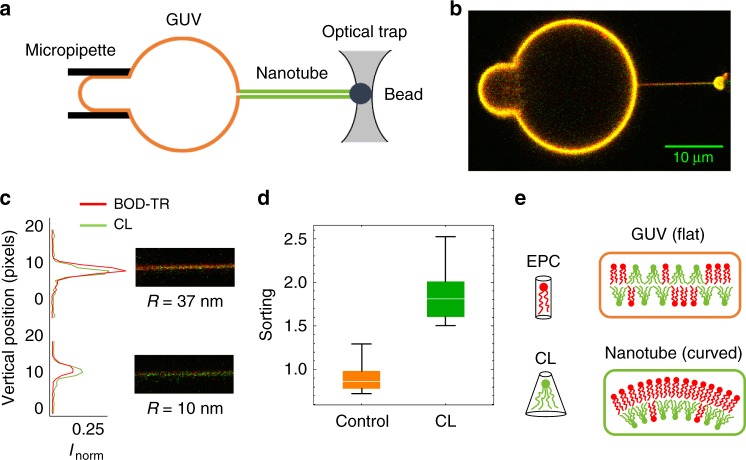
CL is enriched in curved membranes. **a** Schematic of tube assay. A bead in an optical trap is used to pull a membrane tube from a GUV held by a micropipette. The pressure in the micropipette controls the membrane tension and the resulting tube radius. The concentration of the reference lipid and the CL in the tube are measured by confocal fluorescence microscopy. **b** Confocal image of a tube pulled from a GUV containing CL. The membrane (red) was labelled with a fluorescent reference lipid (Bodipy TR-Ceramide) and the CL (green) with Top-Fluor CL. Contrast has been enhanced, and green and red intensities have been scaled to match in the GUV (which is orange-yellowish). The green color of the tube reflects CL enrichment in the tube (relative to the GUV). **c** Images and intensity profiles of tubes pulled from GUVs containing CL for large (*R* ≈ 37 nm) and small (*R* ≈ 10 nm) tube radii. CL is enriched in curved membranes obtaining higher intensity in the green channel compared to the red channel in small tubes. **d** Box plots comparing the sorting ratio for curved tubes (*c* = 0.10 ± 0.03 nm^−1^) pulled from GUVs containing green fluorescent lipids: control (in orange) and a CL density of 0.10 ± 0.05 molecules per nm^2^ (in green). The median is represented with a line; the box plot represents the 25th–75th percentiles; and the error bars show the 5th–95th percentile. CL is enriched in the tubes (average sorting ratio 1.9 ± 0.3, *N* = 10 GUVs) comparing with the lipid control (average sorting ratio 0.9 ± 0.2, *N* = 21 GUVs). (E) CL molecules bend the membrane in the direction of the imposed curvature (inner monolayer, which drives CL enrichment) whereas bends the membrane against the imposed curvature (outer monolayer, which causes CL depletion)

Here, using a fluorescent labelled CL-dye (Top-Fluor CL) with a modified polar head that does not meaningfully increase its intrinsic molecular curvature with respect to bare CL, we measure CL sorting as a function of membrane curvature in a lipid mixture of CL with egg phosphatidylcholine (EPC) at different CL-contents (see Supplementary Methods). Membranes nanotubes of controlled radii are pulled out from GUVs using micromanipulation techniques [see Fig. 1 and Supplementary Methods; also ref. ^31^]. On the one side, a GUV is held by a micropipette exerting a small suction. On the other side, an optically trapped bead is used to apply the force necessary to pull a membrane nanotube. The control of the pipette aspiration sets the tube radius over a biologically relevant range. This construction allows for a quantitative validation of the curvature-induced sorting hypothesis^32^. We have also implemented a model based on membrane elasticity and van der Waals entropy including possible CL–CL interactions. Interestingly, we experimentally found that CL becomes enriched in the tubes, with higher enrichment in the more highly curved membranes. Despite entropic mixing in the lipid mixture, CL sorting is observed up to relatively high CL concentrations, which is plausibly explained by the presence of cohesive CL–CL interaction. This supports the idea for CL self-association into finite-sized molecular clusters, as observed in vivo at highly curved membrane sites^4–8^.

## Results

### Measuring curvature-induced CL sorting in vitro

Membranes nanotubes of controlled radii were pulled out from GUVs made of the biomimetic mixture EPC/CL. Two fluorescent lipid probes were included to detect lipid sorting: green-labelled CL (Top-Fluor CL) and red-labelled reference (Bodipy-TR Ceramide). Since the distribution of this reference dye is approximately uniform in our system^14^, the enrichment of the CL in the tube can be measured by comparing its fluorescence *I*_CL_ to the fluorescence of the Bodipy-TR Ceramide*I*_BOD−TR_. If CL had the same affinity for the membrane tube as Bodipy-TR Ceramide, then their fluorescence intensities would be similar and the tube would appear yellowish. On the contrary, if the CL-to-Bodipy-TR Ceramide ratio in the curved membrane is greater/lower than in the flatter GUV membrane, then the tube would appear greenish/reddish. This relative CL enrichment can be quantified by the sorting ratio, *S*, defined as

1 $$S = \frac{{\left( {I_{{\mathrm{CL}}}/I_{{\mathrm{BOD}} - {\mathrm{TR}}}} \right)_{{\mathrm{tube}}}}}{{\left( {I_{{\mathrm{CL}}}/I_{{\mathrm{BOD}} - {\mathrm{TR}}}} \right)_{{\mathrm{GUV}}}}}\#$$

According to this definition, *S* > 1 implies that CL is enriched in the tube as compared to the GUV, while a value in the range 0 ≤ *S* < 1 means that CL is depleted from the tube. In the absence of driving mechanisms for sorting, the CL molecules must be homogeneously distributed throughout the membrane both in the tube and in the GUV, resulting in a sorting ratio equal to unity, *S* = 1. In our experiments, mesoscopic phase separation was not observed (Supplementary Methods, Supplementary Fig. 1) and dye photobleaching was negligible under our confocal illumination (Supplementary Methods, Supplementary Fig. 2). The sorting ratio is expected to be equal to unity at large tube radii (*R*), when the effect of membrane curvature (*c* ≡ 1/*R*) is negligible and CL molecules must be homogeneously distributed throughout the whole membrane (Supplementary Methods). We studied lipid tubes over a biologically relevant range of radii between 8 and 40 nm (corresponding to curvatures in the range 0.02–0.13 nm^−1^), which are controlled through GUV aspiration (see Fig. 1a–c).

### Curvature induces cardiolipin sorting

Figure 1c shows confocal microscopy images of membrane tubes formed from GUVs containing CL (at a density of about 0.04 molecules per nm^2^). For the thicker tube (*R* ≈ 37 nm), the Bodipy-TR-Ceramide (red) fluorescence signal is similar to that of CL (green) and the tube appears orange-yellowish with a calculated sorting ratio *S* = 0.99 ± 0.01. This means that the lipid composition of the membrane tube is indistinguishable from that of the GUV at low tube curvatures, *c* ≡ 1/*R* = 0.03 nm^−1^. In contrast, when the nanotube radius was reduced down to *R* ≈ 10 nm (*c* = 0.10 nm^−1^), the CL fluorescence (green) in the tube increases with respect to the fluorescence (red) of the Bodipy-TR Ceramide; then, the tube appears greener with a significantly high value of the sorting ratio *S* = 2.19 ± 0.01. An additional control experiment was performed to discard possible systematic differences between the red and green lasers in this set-up. In this control, vesicles did not contain CL but a green fluorescent lipid (BODIPY-FL HPC), which was previously shown to have equal affinity for the membrane tube that the lipid dye emitting in the red channel with Bodipy-TR Ceramide^26^. In this control experiment, the sorting ratio was approximately equal to unity even for curved membranes (*S* = 0.9 ± 0.2), as expected (see Fig. 1d).

**Fig. 2:**
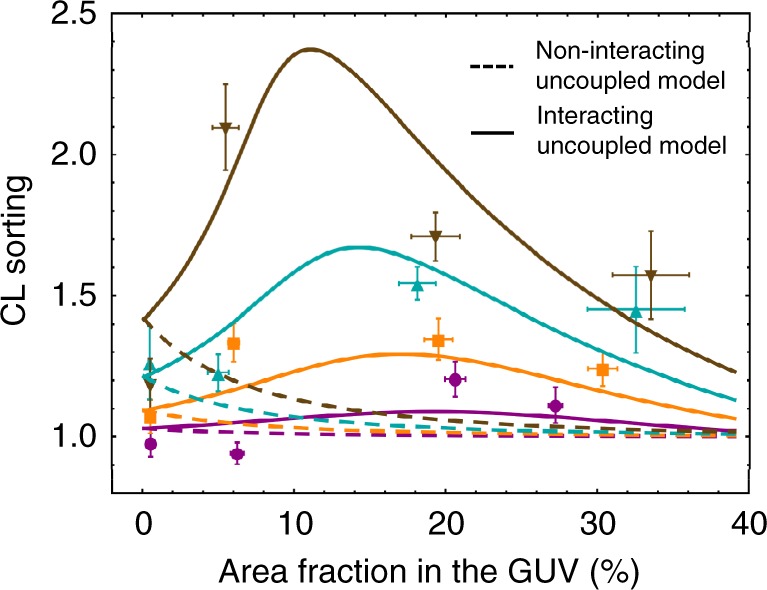
CL enrichment as a function of CL density in GUVs. CL sorting as a function of the area fractioning the GUV *ρ*_GUV,CL_ × *A*_*CL*_ (in percentage) for four ranges of tube curvature:  very high *c* = 0.13 ± 0.02 nm^−1^;  high 0.09 ± 0.02 nm^−1^;  low 0.062 ± 0.012 nm^−1^; and  very low 0.034 ± 0.00 nm^−1^. The points are the arithmetic averages of binned CL GUV densities and sorting ratios, and the error bars represent the measurement error accumulated to the corresponding standard deviations. Dashed lines represent the minimum square fit to the non-interacting uncoupled model (i.e., in the absence of binary interactions between CL molecules, interacting parameter *a* = 0), which gives a CL intrinsic curvature of *c*_*CL*_ = −1.12 ± 0.4 nm^−1^. Solid lines represent the minimum square fit to the interacting uncoupled model (i.e., assuming possible CL–CL interactions with the free interacting parameter a is represented with solid lines and gives ***c***_***CL***_ = −1.10 ± 0.05 nm^−1^ and *a* = (−18 ± 1) *k*_*B*_*T* nm^2^. The computations are made with the following values: CL area^33^ *A*_*CL*_ = 1.3 nm^2^, bending modulus of a pure CL bilayer^33^, this is *κ*_CL_ = 26*k*_*B*_*T* and bending modulus of a pure EPC bilayer^14^, *κ*_EPC_ = 10*k*_*B*_*T*

To determine whether CL sorting depends on membrane density, we prepare GUVs with different CL concentrations *ρ*_GUV,CL_ ranging from approximately 0.004 molecules per nm^2^, which corresponds to a partial area fraction *ρ*_GUV,CL_ × *A*_CL_ of about 0.5% (with the average cross-sectional area of CL, *A*_CL_ = 1.3 nm^2^) up to ~0.25 molecules per nm^2^, which corresponds to an area fraction of ~30% (for details on statistical data binning see Supplementary Notes and Supplementary Tables 1 and 2). Figure 2 shows the CL enrichment as increasing area fraction in four ranges of tube curvature. We find that CL becomes enriched in the tubes, with higher enrichment in more highly curved membranes. Furthermore, our results indicate that CL is enriched in curved membranes at both low and high CL densities with greater sorting at intermediate ones (between 0.1 and 0.15 molecules per nm^2^) (see Supplementary Fig. 4).

### Non-interacting model

Membrane deformations, such as the creation of nanotubes, imply an increase of membrane bending energy. In its simplest form, the bending energy per unit of membrane area can be calculated using the elastic response of a thin sheet:

2 $$g_{\mathrm{bend}} = \frac{1}{2}\kappa \left( {c - c_0} \right)^2,\#$$

where *κ* is the bending modulus, *c* is the membrane curvature, and *c*_0_ is the spontaneous curvature, which permits to describe bilayers that are prone to bend in their equilibrium state due to the compositional inhomogeneity between the inner and the outer monolayers.

Experimental measurements of pure CL bilayers yield a bending modulus of^33^ *κ*_CL_ = 26 *k*_*B*_*T*, which is larger than that observed in pure EPC bilayers^14,34^ *κ*_EPC_ = 10 *k*_*B*_*T*. Since CL molecules stiffen the membrane, we would expect a depletion of CL in the curved tube with respect to the GUV reservoir (*S* < 1). Thus, we hypothesize the observed enrichment (*S* > 1) to be related to spontaneous curvature as a reduction of the bending energy through the intrinsic curvature of the CL. Lipids that compose EPC globally have a nearly cylindrical shape with an intrinsic curvature close to zero (see Fig. 1e). Additionally, the area per molecule of CL is nearly a factor of two larger than that of EPC lipids, due to its four acyl chains as compared to the two of EPC; the average cross-sectional area of EPC^34^ is around 0.69 nm^2^, while it extends out almost twice for CL^33^, around 1.30 nm^2^, which discards possible sorting due to intermonolayer area-difference occurred upon CL concentration in the inner monolayer^35^.

**Fig. 3:**
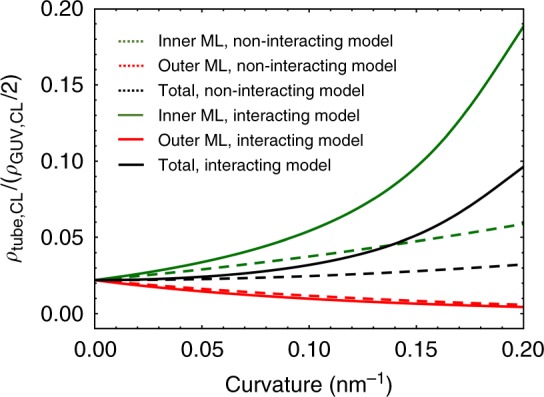
CL density in the nanotube’s monolayers according to the uncoupled model. CL molecules with a negative intrinsic curvature (*c*_*CL*_ = −1.1 nm^−1^) are predicted to be enriched in the inner monolayer (green lines), which bend the membrane in the same sense than the imposed curvature, whereas they are progressively depleted from the outer monolayer (red lines). Unlike the non-interacting model (green dashed line), the interacting model predicts CL enrichment in the inner monolayer of the nanotube (green solid line). The computations are made with the following parametric set (CL area^33^ *A*_*CL*_ = 1.3 nm^2^, bending modulus of a pure CL bilayer^33^ *κ*_CL_ = 26*k*_*B*_*T*, bending modulus of a pure EPC bilayer^14^ *κ*_EPC_ = 10 *k*_*B*_*T*, for a CL density in the GUV of *ρ*_GUV,CL_ = 0.04 molecules per nm^2^, corresponding to an area fraction of about 6% (half in each monolayer)

To implement the sorting model due to spontaneous curvature, CL molecules are assumed to insert homogeneously, but not necessarily at equal amount, in both monolayers with opposite orientations with respect to an external frame of reference; by convention, the curvature is chosen positive for the outer-convex monolayer (*c*^(out)^ > 0), and negative for the inner-concave one (*c*^(in)^ > 0). Then, CL molecules with a given intrinsic curvature *c*_CL_ can be thought as species the elicit a different change in bending energy as placed either in the outer or in the inner monolayer. In case to be endowed with a negative intrinsic curvature (*c*_CL_ < 0), CL molecules contribute either to increase the bending energy if placed in the outer monolayer, or to stabilize the highly curved tube if sorted in the inner monolayer. In general, these opposite effects do not cancel each other, so the total CL concentration of the nanotube varies with curvature, as shown in Fig. 3. Thus, the observed sorting could be related to a reduction of the bending energy through the negative intrinsic curvature of the CL accumulated in the inner monolayer of the lipid tube. At equilibrium, this reduction of bending energy has to be counterbalanced with the entropic cost of distributing molecules non-uniformly (*g*_ent_), which may be approximated with the van de Waals entropy. Finally, the expected sorting is calculated by computing the tube composition that minimizes the total free energy of the tube (*g*_T_ = *g*_bend_ + *g*_ent_) coupled to the nearly flat GUV. This model neglects possible interactions among lipid species thus it will be named below as the non-interacting model. Additionally, two different scenarios are considered: uncoupled or coupled monolayers (Supplementary Notes and Supplementary Figs. 5–9). (1) Uncoupled monolayers (i.e., free to slide past each other), which behave as two independent systems, each one with its own monolayer bending energy. In that case, Eq. (2) is computed with the bending modulus and spontaneous curvature given for the CL abundances in the respective monolayer (Supplementary Fig. 6, Supplementary Notes). (2) In the coupled model, the two monolayers are stuck together, thus the bilayer bends as a uniform whole. In that case, the bending energy is computed for the whole bilayer as a single flexible sheet, with the bending modulus and spontaneous curvature given by the CL abundances in both monolayers (see Supplementary Notes for details).

### CL intrinsic curvature enhances sorting at very low CL densities

At very low CL densities (corresponding to area fractions *ρ*_GUV,CL_ × *A*_CL_ lower than 0.02 with *A*_CL_ = 1.3 nm^2^; see Fig. 2), CL enrichment in the tube is enhanced by the intrinsic curvature contribution and limited by the bending modulus and entropic penalty, as predicted by the non-interacting model (Supplementary Notes). These effects mutually counterbalance and the resulting sorting does not exceed 1.5. Only at this very low CL densities the non-interacting model is enough to explain the observed CL sorting induced by membrane curvature.

### Observed sorting at high CL densities is higher than expected from the non-interacting model

In Fig. 2, the fit of the experimental data to non-interacting uncoupled model is represented with dashed lines. Clearly, the non-interacting model fails to reproduce the experimental data, for any values of the tube curvature (*c*). Although the non-interacting model describes a curvature-increasing sorting at low CL-content, it largely underestimates the results at intermediate and high CL concentration. The non-interacting coupled model also fails to describe the data (Supplementary Notes and Supplementary Fig. 9).

### Interacting model

In general, membranes consist of a mixture of lipids that are characterized not only by elasticity and entropy but also by the short-range interactions among the different lipid species. Conclusions extracted from the previous non-interacting model are strongly altered by including an excess energy per unit of membrane area, which is associated to binary interactions between CL molecules with the form:

3 $$g_{{\mathrm{int}}} = a\rho _{{\mathrm{CL}}}^2\#$$

where the interaction parameter *a* can take positive or negative values depending on whether the CL–CL interactions are repulsive or attractive, respectively. In the complete interacting theory, CL sorting between the nanotube and the vesicle is determined by the tradeoff between bending, mixing entropy, and interaction contributions to the free energy, this is *g*_T_ = *g*_bend_ + *g*_ent_ + *g*_int_ (see Supplementary Notes). It can be obtained numerically by computing the tube composition that minimizes the total free energy of the system. Note that the previous non-interacting model, in the absence of binary interactions, corresponds to a value *a* = 0 (*g*_int_ = 0).

### Observed CL sorting can be explained by the interacting model

Solid lines in Fig. 2 correspond to the best fit of the interacting uncoupled model to the experimental data. Unlike the preceding model (with *a* = 0, dashed lines), the interacting uncoupled model is successful in reproducing the experimental measurements (see solid lines in Fig. 2). The intrinsic curvature of CL (*c*_CL_) is obtained as a parameter of the fit, resulting in a value *c*_CL_ = −1.10 ± 0.05 nm^−1^, in agreement with previous estimates^2–4^. The interaction parameter *a* is also obtained from the fit, resulting in a negative value of *a* = (−18 ± 1) *k*_*B*_*T* nm^2^, which suggests the existence of attractive CL–CL interactions. The interacting uncoupled model predicts an increase of sorting with density (or equivalently with the area fraction *ρ*_GUV,CL_ × *A*_CL_ up to a maximum value from which sorting starts to decrease (see Fig. 2). This reflects accurately the trade-off between bending, entropic, and interaction contributions to free energy that explain the observed CL sorting with a preference in the inner monolayer of the lipid tube (*c*_CL_ ≤ 0). At very low CL densities, the interaction contribution is negligible, thereby there are no appreciable differences between the results of the non-interacting and the interacting models. In this regime, sorting is enhanced by the CL intrinsic curvature contribution and limited by the bending modulus and entropic penalties. As CL density increases, the interaction contribution tends to increase the relative CL enrichment in the tube while the bending and the entropic contributions tend to reduce it. Therefore, the initial increase of sorting with density showed in Fig. 2 does exist because the cohesive CL–CL interaction offsets both the bending and the entropic penalties, causing CL accumulation in the inner (coherently curved) monolayer (see Fig. 3). From certain onset in CL density the cohesive interaction contribution becomes lower than contributions by bending and entropy, thus sorting starts to decrease (for a detailed comparison of these contributions to free energy, Supplementary Notes and Supplementary Figs. 7 and 8). In addition to the uncoupled sorting model, we have also performed the fit to a membrane model assuming monolayer coupling within the membrane bending energy. Although the interacting coupled model also predicts high sorting at high CL densities, it fails in reproducing the observed sorting at high curvatures (see Supplementary Fig. 9). Taken together, the theoretical analysis to our experimental data suggests the existence of short-range CL–CL attractive interactions and the uncoupled behavior of EPC/CL bilayers. This result endows both geometry-coherent lateral packing of lipids and the additional lateral CL–CL cohesion as the dominant factors of curvature mediated CL sorting in lipid tubes.

Finally, it is important to verify whether CL molecules can exchange between the GUV and the tube. This test is essential since a diffusion impediment would mean that the CL distribution in our experiments would not be at equilibrium. To study this CL transport, we first photobleached the fluorescently-labelled CL contained in the tube by imaging it at high laser power. Next, we monitored the green fluorescence recovery after photobleaching by imaging the tube at low laser power. Our observations establish that CL repopulates the tube in a few seconds after bleaching (see Supplementary Fig. 2), deducing that there is no detectable diffusion barrier at the tube-neck. In a more biological wisdom, this result depicts CL as a mobile molecule, and the association clusters formed by CL–CL interactions as labile structures in dynamic equilibrium with their lipid environment.

## Discussion

Despite evidences from in vivo studies, and the results from MD simulations showing that CL concentrates in negatively curved regions of membranes, there was no quantitative data that support the hypothesis for a curvature-based CL enrichment. Using an in vitro approach with a homogenous mixture of EPC and CL, we have seen that membrane shape alone can modulate the distribution of CL without the involvement of any cellular protein machinery. We have found that CL molecules with a cone-like shape accumulate in curved regions of the membrane, with higher sorting in more highly curved membranes and preferred accumulation in negatively curved sites. A negative value for the intrinsic curvature of the CL molecule (*c*_CL_ = −1.1 nm^−1^) is raised from the analysis of the experimental data in view of a theory of spontaneous curvature with lipid interactions. The calculated value for CL is compatible with previous estimates^2,3^, being in quantitative agreement with direct measurements of membrane curvature in nano-vesicles formed by other cone-shaped lipids^36^. In general, the amount of lipid sorting arises from a competition between two forces: the energetic advantage of curvature matching and the cost of mixing entropy. For single lipids, previous works showed an energetic gain as low as 1% of *k*_*B*_*T* at physiological curvatures^23,30^, which suggests that geometry alone does not contribute to sorting due to the overwhelming cost of mixing entropy at the cellular scale. Using computational approaches, Huang et al. have shown that this conclusion can be critically altered provided lipid-lipid interactions are considered through stable finite-sized lipid clusters, which can spontaneously and independently target the lipids to curved regions of the membrane^30^. Their theoretical analysis agrees with our result that CL–CL attractive interactions are required to explain the observed sorting. In an experimental monolayer study, Sennato et al.^37^ reported differences in the molecular interactions between different lipids indicating that CL interacts repulsively with PE and PC, the other two main lipid components of the mitochondrial inner membrane. Therefore, the predicted CL–CL interactions are expected to induce the formation of self-associating CL finite-sized clusters possibly stabilized by long-range repulsive electrostatic interactions^16^. These opposing forces should determine the CL cluster size that stabilize curvature in vivo. Model fits to our data predict an interaction parameter *a* ≈ −18 *k*_*B*_*T* nm^2^; since *A*_CL_ = 1.3 nm^−2^ and *c*_CL_ = −1.1 nm^−1^, using the results of ref. ^30^ we get an estimate for the ratio of the interaction energy to the intrinsic curvature squared of $a/\left( {A_{{\mathrm{CL}}}c_{{\mathrm{CL}}}^2} \right) \approx - 11$ *k*_*B*_*T*/nm^−2^, which gives a cluster size of the order of 10 molecules. The CL cluster size obtained (≈10 × *A*_CL_ ≈ 10–20 nm^2^) resembles the nanometric estimate of lipid-raft dimensions^37^. However, no visible evidence of phase separation in EPC/CL bilayers was obtained under the confocal microscope, neither in the tubes nor the mother GUVs. Taking into account that fluorescence microscopy only detects mesoscopic lipid domains (larger than 1 µm), and despite the full miscibility of mixed EPC/CL monolayers observed in flat monolayers^16^, the existence of nanosized domains in curved bilayers cannot be discarded. Thus, definitive evidences might be obtained from CL-containing bilayers in highly curved tubes, an experimental setting extremely difficult to be realized with AFM or other nanoscopies that allow to resolve molecular clusters. Here, we have unequivocally determined that CL molecules can be sorted by curvature into clusters. This is biologically relevant results that contribute to understand the interplay between CL composition and membrane shape^38,39^.

## Material and methods

### GUVs formation

GUVs made of EPC and CL were grown by using electroformation technique (22 ± 1 °C, 400 mOsm sucrose solution; see Section 1 in Supporting Information for details). To allow membrane visualization, we used the red fluorescent reference lipid, BODIPY-TR Ceramide (1% mol/mol). The green fluorescent lipid, Top-Fluor CL, is also included (1% mol/mol) to allow the quantification of the CL-enrichment. To favor adhesion between the GUV membrane and the streptavidin-coated beads holding the tube in the trap, DSPE-PEG(2000)-Biotin (0.2% mol/mol) was added to the electroformation mixture. The green fluorescent lipid BODIPY-FL HPC (1% mol/mol) was used for control experiments (no CL), and for the green fluorescence calibration (see Supplementary Method 3E and Supplementary Fig. 4).

### Procedure for membrane nanotube extraction

The procedure is based on pulling membrane nanotubes from GUVs aspirated in a micropipette (see ref. ^31^ for details). In every experiment, a GUV is aspirated in a micropipette and a streptavidin-coated bead is trapped in an optical trap. The GUV, which contains a very small fraction of biotinylated lipids, is then pushed against this bead so that a small patch of membrane sticks to the bead. The vesicle is then pulled away to create a membrane nanotube of 5−10 μm in length.

### Statistics and reproducibility

In order to adequate statistics, for every specimen studied we perform successive step-variations of the membrane tension (which changes the tube radius *R*) by adjusting the pipette aspiration pressure. The tube radius was determined by comparing the red fluorescence intensity of the membrane tube to that of the GUV through a previous calibration with the reference lipid BODIPY-TR Ceramide (see Supplementary Method 3D). We await at least 45 seconds between successive readouts to let the system equilibrate by lipid diffusion. Then, the fluorescence image is acquired using a confocal microscope. At least *N* = 6 replicas, typically *N* ≥ 10, were performed at each experimental condition considered in our rationale (see Supplementary Notes). All experiments were performed at room temperature, 22 ± 1 °C.

### Reporting summary

Further information on research design is available in the Nature Research Reporting Summary linked to this article.

## Supplementary information

Supplementary Information

Reporting Summary

## Data Availability

All data generated or analyzed during this study are included in this published article (and its Supplementary information files).
