# Supplementary material for: Membrane curvature induces cardiolipin sorting
Source: Commun Biol. 2019 Jun 20;2:225. doi: 10.1038/s42003-019-0471-x (PMC6586900; doi:10.1038/s42003-019-0471-x)
Supplement: Supplementary file 1 — Supplementary Information [file 42003_2019_471_MOESM1_ESM.pdf]

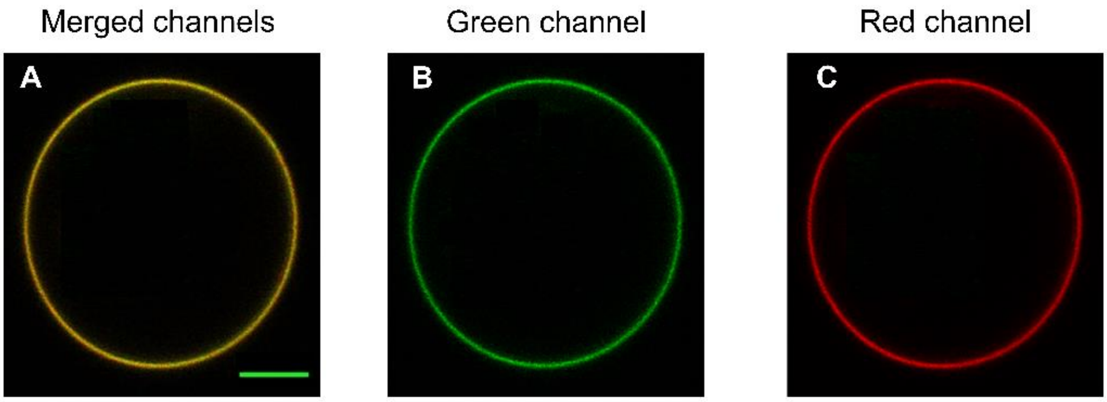

Supplementary figure 1. Absence of phase separation. Confocal image of a GUV with a total CL concentration of 40% by mole showing the distribution of Top-Fluor CL (green channel) and Bodipy-TR Ceramide (red channel). There is no visible evidence of phase separation in EPC/CL bilayers but GUVs exhibit a homogeneous fluorescence along their contour. Scale bar is 5 µm.

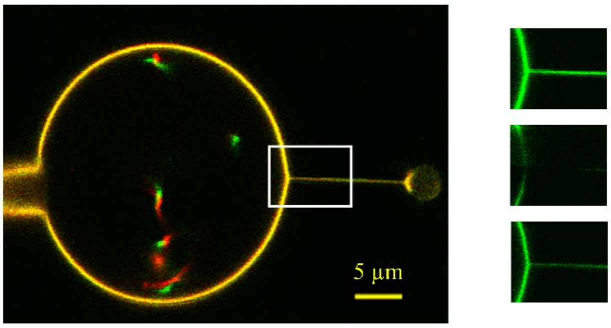

Supplementary figure 2. CL molecules can diffuse across the membrane nanotube. CL fluorescence recovery in the tube ($R = 25$ nm, $L = 7$ µm) after photobleaching the zone indicated by the white square. Right: images of the tube before (top); just right after photobleaching (middle) and 6 seconds after photobleaching (bottom).

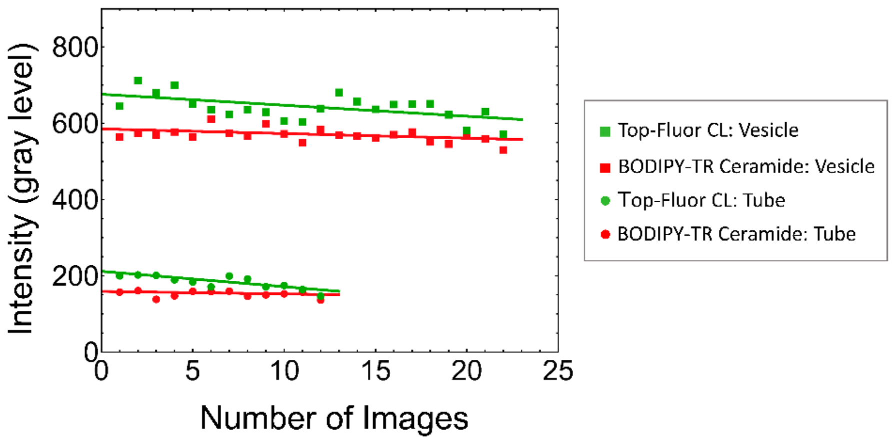

Supplementary figure 3. Photobleaching quantification. Evolution of fluorescence in the vesicle (red and green squares) and in the tube (red and green circles) during the acquisition of consecutive images. Frame rate around 1 Hz. Linear fit to the dataset gives for Top-Fluor CL in the vesicle, $y = 676 - 2.9x$; for Bodipy-TR Ceramide in the vesicle, $y = 585 - 1.2x$; for Top-Fluor CL in the tube, $y = 212 - 4.0x$; for Bodipy-TR Ceramide in the tube, $y = 159 - 0.66x$.

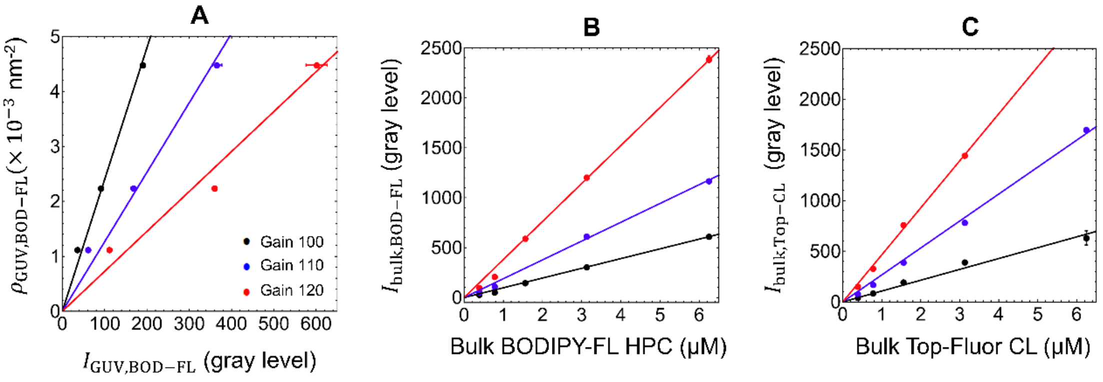

Supplementary figure 4. Determination of CL density in GUVs. Green fluorescence calibration for the three values of PMT gains used during experiments (100 in black, 110 in blue, and 120 in red). (A) Fluorescence measured from GUVs with increasing amount of BODIPY-FL HPC. Linear fit $\rho_{\text{GUV,BOD-FL}} = A^{\text{gain}} \times I^{\text{gain}}_{\text{GUV,BOD-FL}}$ gives the conversion constants $A^{100} = 23.9 \pm 0.9$, $A^{110} = 12.6 \pm 0.7$, and $A^{120} = 7.2 \pm 0.5$. (B) Fluorescence measured in bulk as a function of the volume concentration of BODIPY-FL. (C) Fluorescence measured in bulk as a function of the volume concentration of Top-Fluor CL. The efficient ratio $F^{\text{gain}} = I^{\text{gain}}_{\text{bulk,Top-CL}} / I^{\text{gain}}_{\text{bulk,BOD-FL}}$ can be deduced from the ratio between the slopes of the linear fits $I^{\text{gain}}_{\text{bulk,Top-CL}}$ versus $\rho_{\text{bulk,Top-CL}}$ (C) and $I^{\text{gain}}_{\text{bulk,BOD-FL}}$ versus $\rho_{\text{bulk,BOD-FL}}$ (B) obtaining $F^{100} = 1.10 \pm 0.06$, $F^{110} = 1.41 \pm 0.04$, and $F^{120} = 1.22 \pm 0.03$.

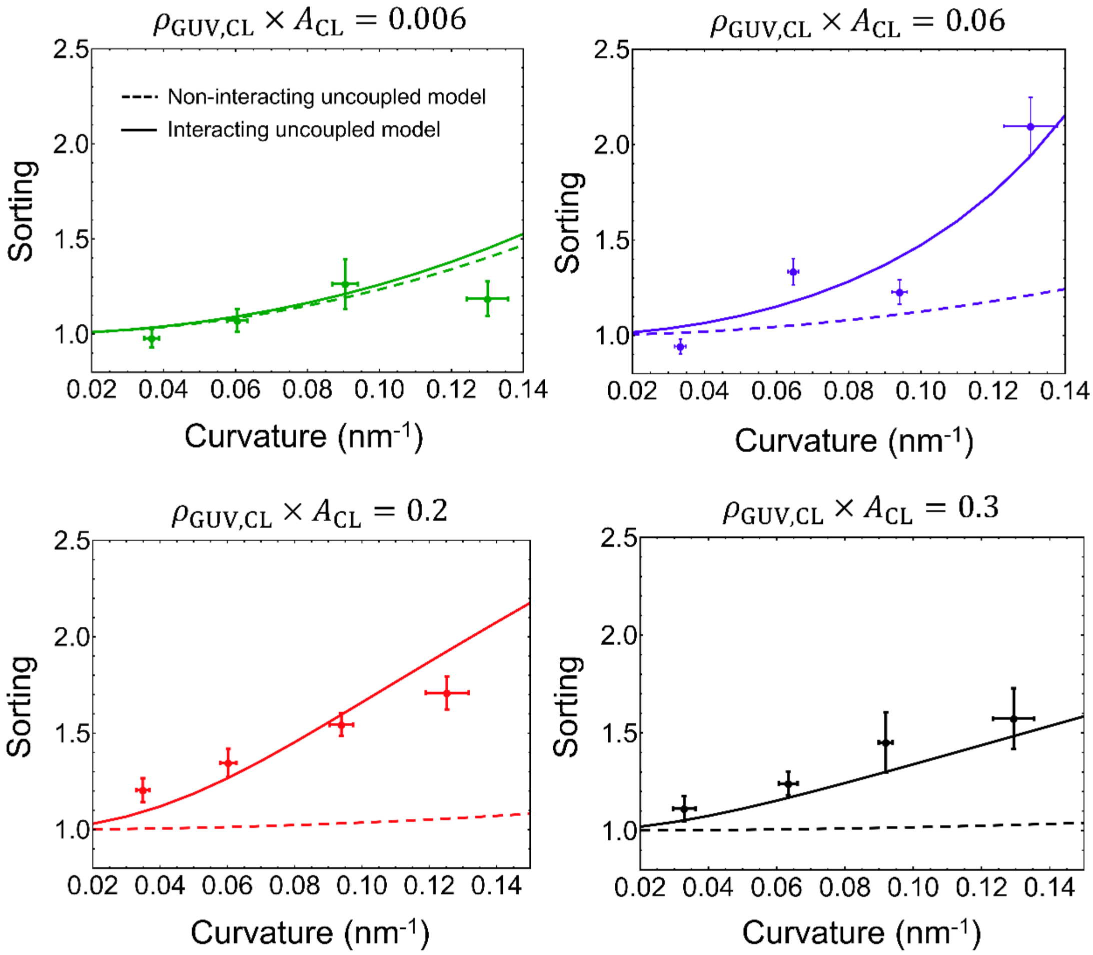

Supplementary figure 5. CL enrichment as a function of tube curvature. CL sorting as a function of the tube curvature for four ranges of the area fraction $\phi = \rho_{\mathrm{GUV,CL}} \times A_{\mathrm{CL}}$: ▬ very low $\phi = 0.0056 \pm 0.0003$; ▬ low $\phi = 0.057 \pm 0.003$; ▬ high $\phi = 0.196 \pm 0.005$; and ▬ very high $\phi = 0.303 \pm 0.008$. Points are the mean of binned tube curvatures and sorting ratios and the error bars represent the corresponding standard deviations. The minimum square fit of the theoretical uncoupled model (*i.e.*, in the absence of binary interactions between CL molecules, $a = 0$) is represented with dashed lines and gives an intrinsic curvature of CL of $c_{\mathrm{CL}} = -1.12 \pm 0.4$ nm^{-1}. The minimum square fit of the interacting uncoupled model (*i.e.*, assuming possible CL-CL interactions, with free interacting parameter a) is represented with solid lines and gives $c_{\mathrm{CL}} = -1.10 \pm 0.05$ nm^{-1} and $a = (-18 \pm 1)\, k_B T$ nm^2. It is clear that the non-interacting uncoupled model does not reproduces our data. In contrast, CL-CL attractive interactions can explain the observed sorting. The computations are made with the following values: CL area[14] $A_{\mathrm{CL}} = 1.3$ nm^2, bending modulus of a pure CL bilayer[14] $\kappa^{\mathrm{b}}_{\mathrm{CL}} = 26\, k_B T$, and bending modulus of a pure EPC bilayer[15] $\kappa^{\mathrm{b}}_{\mathrm{EPC}} = 10\, k_B T$.

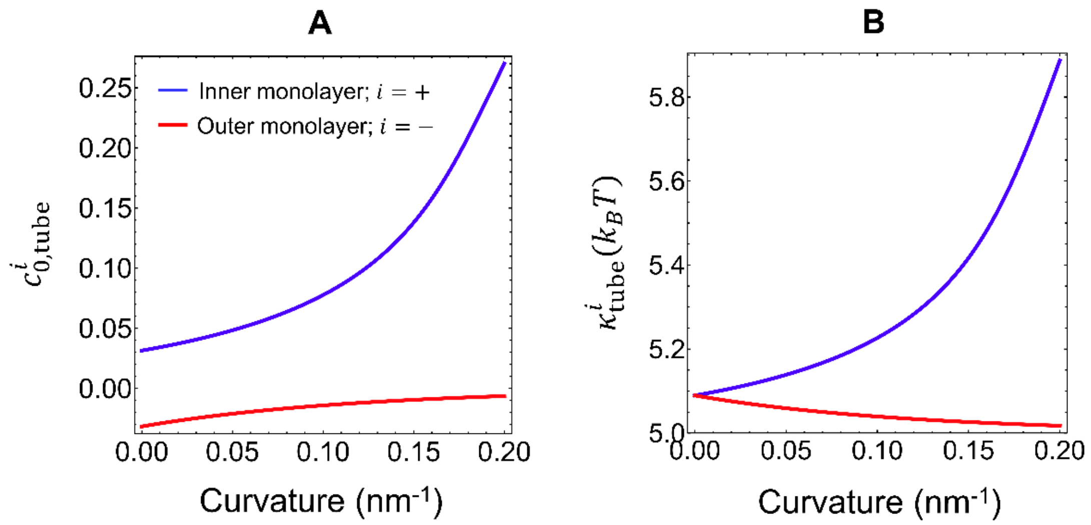

Supplementary figure 6. Spontaneous curvature and bending modulus in the membrane nanotube according to the interacting uncoupled model. (A) The spontaneous curvature in the tube is positive in the inner monolayer and negative in the outer one. It increases with tube curvature in both monolayers due to the enrichment of positively curved CL molecules and the depletion of negatively curved CL molecules. (B) Because CL stiffens the membrane, the bending modulus of the tube increase with curvature in the inner monolayer while decrease in the outer one. The computations are made with the following values: CL area[14] $A_{\text{CL}} = 1.3$ nm^2, bending modulus of a pure CL bilayer[14] $\kappa^{\text{b}}_{\text{CL}} = 26\, k_B T$, bending modulus of a pure EPC bilayer[15] $\kappa^{\text{b}}_{\text{EPC}} = 10\, k_B T$, and a CL density in the GUV of $\rho_{\text{GUV,CL}} = 0.04$ molecules per nm^2, corresponding to an area fraction $\phi = \rho_{\text{GUV,CL}} \times A_{\text{CL}}$ of about 6% (half in each monolayer). Quantities referred to the inner/outer monolayer of the tube are represented with blue/red lines.

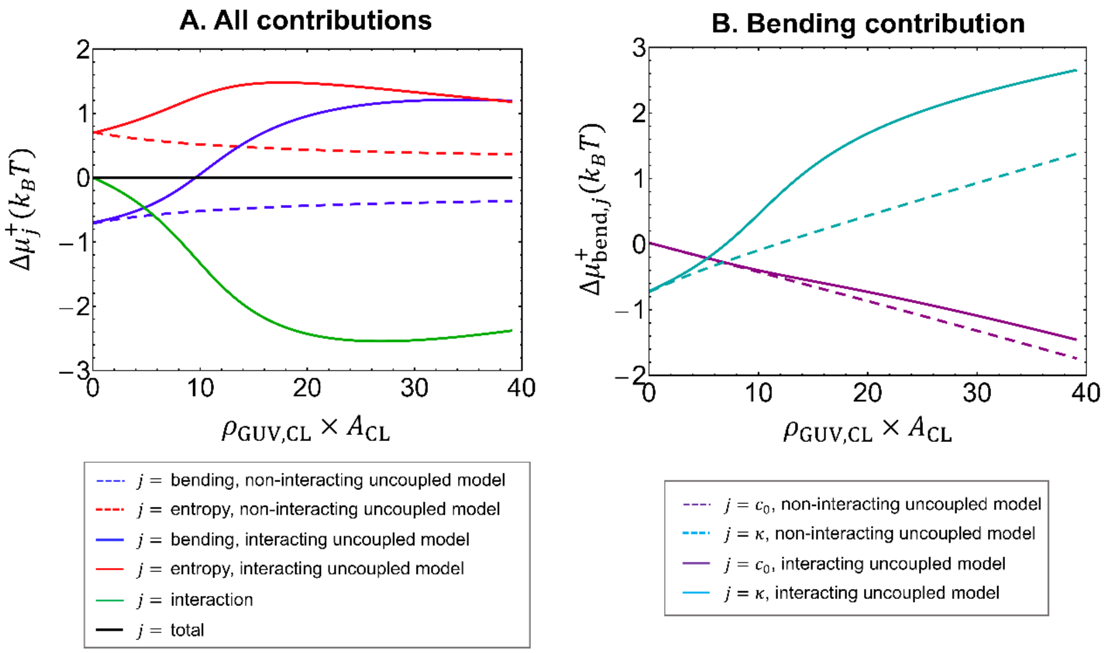

Supplementary figure 7. Bending, entropic, and interaction contributions to sorting as a function of area fraction in the GUV (in percent). In both interacting (solid lines) and non-interacting (dashed lines) uncoupled models, sorting is limited by the entropic term ($\Delta\mu_{\mathrm{ent}}^+ > 0$, red lines). The bending contribution to sorting is, however, quite different between the interacting and the non-interacting uncoupled models: when non-CL interaction is assumed, the bending term (blue dashed line) contributes slightly to sorting ($\Delta\mu_{\mathrm{bend}}^+ \lessapprox 0$) and this contribution diminishes as CL density increases. In contrast, when CL-CL interactions are considered, the bending term enhances CL enrichment just for very low CL densities (blue solid line). Above this value, the bending term drives CL depletion ($\Delta\mu_{\mathrm{bend}}^+ > 0$). The resulting bending contribution to sorting is enhanced by the spontaneous curvature term ($\Delta\mu_{\mathrm{bend},c_0}^+ < 0$, purple lines) but limited by the stiffness penalty ($\Delta\mu_{\mathrm{bend},\kappa}^+ > 0$, cyan lines). The interaction term (green solid line) drive strong CL enrichment ($\Delta\mu_{\mathrm{int}}^+ < 0$) and then it is required to explain the observed data at high densities. Finally, $\Delta\mu_{\mathrm{total}}^+ = 0$ (black line) since the system is at equilibrium. The computations are made with the following values: CL area $A_{\mathrm{CL}} = 1.3\ \mathrm{nm}^2$,[14] bending modulus of a pure CL bilayer $\kappa_{\mathrm{CL}}^{\mathrm{b}} = 26\ k_BT$[14], bending modulus of a pure EPC bilayer $\kappa_{\mathrm{EPC}}^{\mathrm{b}} = 10\ k_BT$[15], and tube curvature $c = 0.1\ \mathrm{nm}^{-1}$.

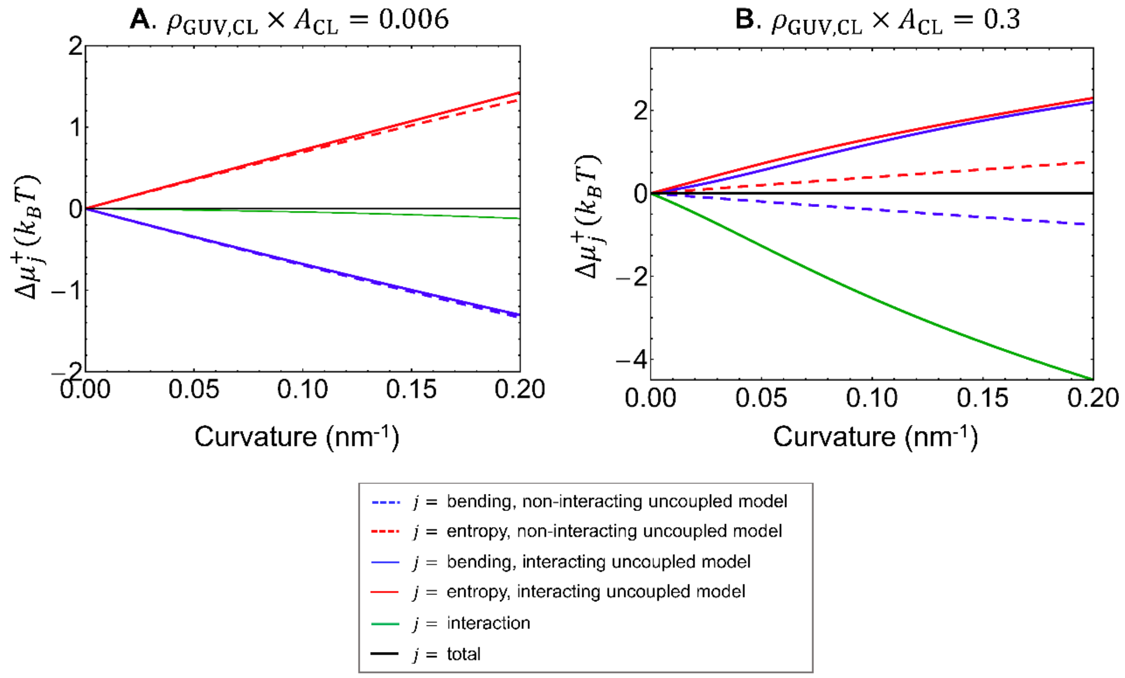

Supplementary figure 8. Bending, entropic, and interacting contributions to sorting as a function of curvature. At low CL densities (A) the influence of CL interaction (green solid line) is negligible, thereby there are no significant differences between the results of the non-interacting (dashed lines) and the interacting (solid lines) models. Entropy drives CL depletion ($\Delta\mu_{\text{ent}}^+ > 0$) and bending contributes slightly to sorting ($\Delta\mu_{\text{bend}}^+ < 0$). In contrast, at high densities (B), there exist quite differences between the interacting and the non-interacting uncoupled models and CL-CL attractive interactions are essential to explain the observed data. The computations are made with the following values: CL area $A_{\text{CL}} = 1.3$ nm^2,[14] bending modulus of a pure CL bilayer $\kappa_{\text{CL}}^{\text{b}} = 26\, k_B T$[14], and bending modulus of a pure EPC bilayer $\kappa_{\text{EPC}}^{\text{b}} = 10\, k_B T$[15].

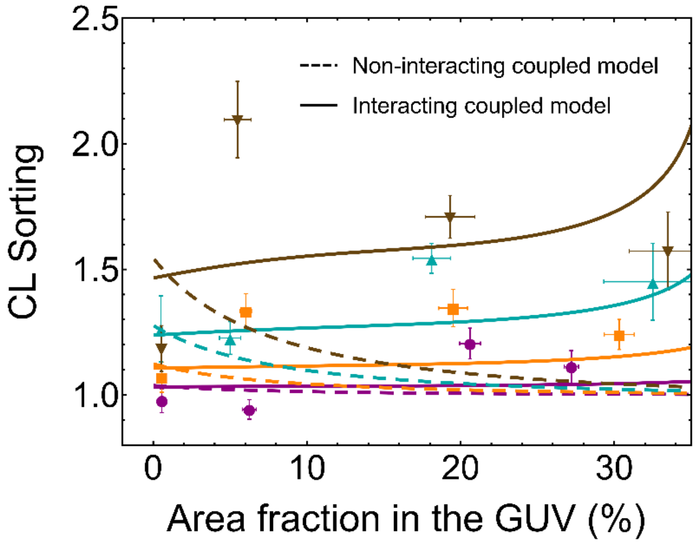

Supplementary figure 9. CL enrichment as a function of CL density in GUVs according to the coupled model. CL sorting as a function of the area fraction $\phi = \rho_{\mathrm{GUV,CL}} \times A_{\mathrm{CL}}$ (in percentage) for four ranges of tube curvature: ▼ very high (0.1287 ± 0.0012) nm^{-1} in brown; ▲ high (0.093 ± 0.008) nm^{-1} in cyan; ■ low (0.062 ± 0.010) nm^{-1} in orange; and ● very low (0.034 ± 0.009) nm^{-1} in purple. The points are the arithmetic means of binned CL GUV densities and sorting ratios, and the error bars represent the corresponding standard deviations. Dashed lines represent the minimum square fit to the non-interacting coupled model (*i.e.*, in the absence of binary interactions between CL molecules, interacting parameter $a = 0$), which gives a CL intrinsic curvature of $c_{\mathrm{CL}} = -0.7 \pm 0.2\ \mathrm{nm}^{-1}$. Solid lines represent the minimum square fit to the interacting coupled model (*i.e.*, assuming possible CL-CL interactions with the free interacting parameter a) is represented with solid lines and gives $c_{\mathrm{CL}} = -0.6 \pm 0.1\ \mathrm{nm}^{-1}$ and $a = (-9.4 \pm 0.4)\ k_B T\ \mathrm{nm}^2$. The computations are made with the following values: CL area $A_{\mathrm{CL}} = 1.3\ \mathrm{nm}^2$,[14] bending modulus of a pure CL bilayer $\kappa^{\mathrm{b}}_{\mathrm{CL}} = 26\ k_B T$[14], and bending modulus of a pure EPC bilayer $\kappa^{\mathrm{b}}_{\mathrm{EPC}} = 10\ k_B T$[15].

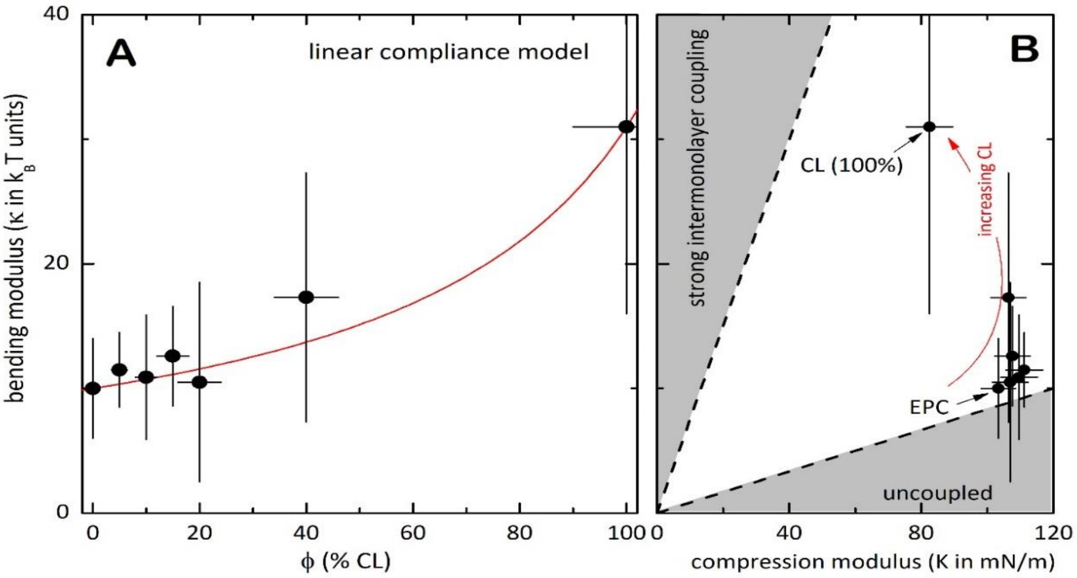

Supplementary figure 10. Mechanical parameters of the EPC + CL system. A) Experimental values of the bending modulus measured by fluctuation spectroscopy as a function of the area fraction $\phi = \rho_{\mathrm{GUV,CL}} \times A_{\mathrm{CL}}$ (in percentage). The red line represents the theoretical prediction from the linear compliance model (Eq. S13). B) Coupling diagram plotted as a correlation between the bending modulus (κ) and the compression modulus (K), as measured in Langmuir monolayers. The regimes (strong coupling/weak coupling) are determined by Eq. (S36).

Supplementary table 1. CL sorting as a function of tube curvature at different CL densities as sorted from statistical data binning (data plotted in Figure 2). Every datasheet is headed by a descriptor of its characteristic CL content, which is expressed as the average value of CL density calculated over all the specimens considered in the table.

Very high density $\rho_{\mathrm{GUV,CL}} = (23.3 \pm 0.6) \times 10^{-2}\ \mathrm{nm}^{-2}$

**Nº of GUVs**	**Curvature** (nm^{-1})	**GUV density** $(10^2\ \rho_{\mathrm{GUV,CL}}\mathrm{in\ nm}^{-2})$	**Sorting**
10	0.033 ± 0.007	20.9 ± 0.4	1.11 ± 0.06
14	0.063 ± 0.012	23.3 ± 0.7	1.24 ± 0.06
6	0.09 ± 0.02	25 ± 2	1.45 ± 0.15
6	0.13 ± 0.03	25 ± 2	1.57 ± 0.15

High density $\rho_{\mathrm{GUV,CL}} = (15.1 \pm 0.4) \times 10^{-2}\ \mathrm{nm}^{-2}$

**Nº of GUVs**	**Curvature** (nm^{-1})	**GUV density** $(10^2\ \rho_{\mathrm{GUV,CL}}\mathrm{in\ nm}^{-2})$	**Sorting**
18	0.035 ± 0.007	15.9 ± 0.5	1.20 ± 0.06
12	0.060 ± 0.012	15.0 ± 0.7	1.35 ± 0.07
8	0.09 ± 0.02	13.9 ± 0.9	1.54 ± 0.06
6	0.13 ± 0.02	14.9 ± 1.2	1.71 ± 0.09

Low density $\rho_{\mathrm{GUV,CL}} = (4.4 \pm 0.2) \times 10^{-2}\ \mathrm{nm}^{-2}$

**Nº of GUVs**	**Curvature** (nm^{-1})	**GUV density** $(10^2\ \rho_{\mathrm{GUV,CL}}\mathrm{in\ nm}^{-2})$	**Sorting**
29	0.033 ± 0.006	4.8 ± 0.3	0.94 ± 0.04
31	0.065 ± 0.012	4.6 ± 0.3	1.33 ± 0.07
12	0.094 ± 0.015	3.8 ± 0.5	1.23 ± 0.06
6	0.13 ± 0.02	4.2 ± 0.7	2.10 ± 0.15

Very low density $\rho_{\mathrm{GUV,CL}} = (0.43 \pm 0.02) \times 10^{-2}\ \mathrm{nm}^{-2}$

**Nº of GUVs**	**Curvature** (nm^{-1})	**GUV density** $(10^2\ \rho_{\mathrm{GUV,CL}}\mathrm{in\ nm}^{-2})$	**Sorting**
23	0.037 ± 0.007	0.43 ± 0.03	0.98 ± 0.05
9	0.060 ± 0.012	0.42 ± 0.05	1.07 ± 0.06
5	0.09 ± 0.02	0.39 ± 0.06	1.26 ± 0.13

6	0.13 ± 0.03	0.41 ± 0.02	1.19 ± 0.09

Supplementary table 2. Control experiment. BODIPY-FL HPC sorting as a function of tube curvature.

Nº of GUVs	Curvature (nm^{-1})	Sorting
14	0.034 ± 0.006	1.03±0.03
7	0.065 ± 0.012	0.93±0.03
6	0.09 ± 0.02	1.04±0.06
6	0.12 ± 0.02	1.00±0.12

Supplementary Methods

GUV Formation

Reagents. Egg-PC (EPC), 18:1 CL, Top-Fluor CL, and DSPE-PEG (2000)-Biotin were purchased in powder form from Avanti Polar Lipids. BODIPY-TR Ceramide (red fluorescent lipid) and BODIPY-FL HPC (green fluorescent lipid) were purchased from Molecular Probes. Streptavidin-coated polystyrene beads with diameter 3-3.4 μm were purchased from Bangs Laboratories (Carmel, IN). β-casein from bovine milk, mineral oil, and other reagents were purchased from Sigma.

Experimental procedure. Lipid mixtures composed of EPC and CL at different CL-content, 4%, 9%, 14%, and 24% by mole, were supplemented with the green fluorescent lipid Top-Fluor CL at 1% by mole to allow the quantification of the CL-enrichment as a function of the membrane curvature (resulting in a total CL concentration of 0%, 5%, 10%, 15%, and 25% by mole, respectively), with the red fluorescent reference lipid BODIPY-TR Ceramide at 1% by mole to allow membrane visualization, and with 0.2% by mole of DSPE-PEG(2000)-Biotin to allow adhesion between the membrane and the streptavidin-coated beads holding the tube in the trap. For control experiments (with no CL) and for the green fluorescence calibration (see Section A6.3 below), we use 1% by mole of the green fluorescent lipid BODIPY-FL HPC. Briefly, the procedure for preparing GUVs using the electroformation technique[1] was the following: two lipid films at 1 mg/ml are spread on two conductive ITO coated glasses and dried under high vacuum during 1 hour. The lipid films are then rehydrated in a sucrose solution 400 mM (osmolarity 400 mOsm) and grown during at least 1 hour under an AC electric field of typically 1.1 V, 10 Hz between the two ITO electrodes. After growth, vesicles were transferred for observation and manipulation in a chamber containing 20 mM Tris pH 7.5 and 200 mM KCl (osmolarity ≈ 400 mOsm). Phase separation was not observed (even at high CL excess, see Supplementary figure 1) but GUVs exhibit a homogeneous fluorescence intensity. All steps were performed at room temperature, 22 ± 1 °C.

Protocol for Tube Pulling Experiments

Tube pulling experiments were performed using a setup developed by P. Bassereau's group. It consists on a commercial Nikon TE2000 confocal microscope equipped with micromanipulators and optical trap. The details of the microscopy setup can be found in Ref. (2) This system allows for manipulating individual GUVs, controlling their surface tension, generating membrane nanotubes, measuring their radius and pulling force, and determining the density of membrane components between the GUV and the tube. The procedure is based on pulling membrane nanotubes from GUVs aspirated in a custom micropipette of ≈ 5 μm diameter. For each experiment, an open micromanipulation chamber was built with two clean glass coverslips (bottom: 11x32 mm, top: 9x35 mm). Before starting experiments, the micropipette and the chamber are filled with a solution containing of β-casein at 5 mg/ml during approximately 20 minutes in order to prevent the GUV from sticking to the glass. Then, the chamber is rinsed with the experimental buffer, containing 20 mM Tris pH 7.5 and 200 mM KCl and then is filled with a few μl of GUVs and streptavidin-coated beads and completed with buffer. To be able to impose a tension using the micropipette aspiration technique it is necessary to have GUVs with

an initially low tension. This can be achieved by waiting for at least 30 min to slowly increase the osmolarity of the solution in the chamber by evaporation. When the tension is low enough (based on the visual observation of fluctuating vesicles), we close both edges of the chamber with mineral oil in order to maintain a constant pressure inside the chamber. Before each set of experiments on a new vesicle, it is necessary to set the zero reference pressure in the micropipette by detecting the absence of movement of a bead in the pipette. Once that is done, a GUV is aspirated in a micropipette applying a high aspiration pressure (checking the GUV "tongue" in the micropipette) and a streptavidin-coated bead is trapped in an optical trap. The vesicle, which contains a very small fraction of biotinylated lipids, is then pushed against this bead so that a small patch of membrane sticks to the bead. The vesicle is then pulled away to create a membrane nanotube of 5-10 µm in length. This length is kept constant during the whole experiment. We perform successive step-variations of the membrane tension (which varies the tube radius R over a biologically relevant range, typically between 8 and 40 nm) by adjusting the pipette aspiration pressure. Between each data point we wait for at least 45 seconds to let the system equilibrate by lipid diffusion (enough time to ensure that the system is at equilibrium, see Supplementary figure 2) and then we acquire one fluorescence image. All experiments were performed at room temperature, 22 ± 1 ºC.

Fluorescence Quantification

The confocal images are acquired with the EZ-C1 software (Nikon) and then analyzed using a custom Matlab program which allows to quantify the intensity values for the two fluorophores in the tube and in the vesicle, as described previously in Ref. (3). The software works within a user-defined regions of interest (ROIs). In our case, ROIs are delimited along the GUV contour and along the tube. Within these regions, pixel intensity values are averaged along horizontal lines, converting the images into one-dimensional data sets. After that, the maxima of the fluorescence intensity profiles are reduced by the corresponding noise levels (average values of the background), which are typically lower than 5% of average fluorescence. The reduced fluorescence intensities are used to determine the CL density in GUVs, the sorting ratio, and the tube radius. The same area in the tube and in the vesicle is used for each dye channel.

Absence of phase separation. Under our confocal image acquisitions conditions, we observed no phase separation but GUVs exhibit a homogeneous fluorescence along their contour. Supplementary figure 1 shows a confocal image with a total CL concentration of 40% by mole. Even at this high CL excess, there is no visible evidence of phase separation in EPC/CL bilayers.

Photobleaching. Under our imaging conditions (laser intensity, pixel dwell, pinhole size and PMT gain), photobleaching is weak. Our confocal microscopy is designed to acquire sequentially the green and the red channel images in order to avoid any fluorescence bleed between both channels. We can estimate the amount of photobleaching by fitting the fluorescence decrease with the number of consecutive images (see Supplementary figure 3). In the vesicle, the loss of intensity per image is less than 0.2% for BODIPY-TR Ceramide (red channel) and less than 0.4% for Top-Fluor CL (green channel). The bleaching of the tube, which is larger than the vesicle due to its confined geometry, results in a loss of intensity per image less than 0.4% for BODIPY-TR Ceramide (red channel) and less than and 2% for Top-Fluor CL (green channel), respectively. To further limit bleaching, only one image per membrane tension

step is acquired and no more than 8 images are taken during one experiment on the same vesicle.

Sorting ratio. The CL-enrichment relative to the reference lipid (Bodipy-TR-Ceramide) is quantified by the sorting ratio S. We calculate it as the ratio of fluorescence of green and red channels in the tube normalized by the same ratio in the vesicle

$$S = \frac{(I_{\text{CL}}/I_{\text{BOD-TR}})_{\text{tube}}}{(I_{\text{CL}}/I_{\text{BOD-TR}})_{\text{GUV}}} \#(\text{S1}) \qquad \#$$

The control experiment is made with vesicles containing no CL but 1% by mole of the green fluorescent lipid BODIPY-FL HPC. Our fluorescence labelled lipids (Bodipy-TR Ceramide, Top-Fluor CL, and Bodipy FL-HPC) are not sensitive to membrane polarization and then we have not to correct for the polarization effects. We can conclude this from the measurements on membrane tubes with very low curvatures (*i.e.*, large radii), where we expect the sorting ratio to be equal to unity independently of membrane composition. As shown in Tables S1 and S2, the sorting ratio approaches 1 at very low curvatures, and then Eq. (S1) requires no normalization factor. For control experiments the observed sorting is approximately equal to one even for curved membranes, as expected (see Supplementary table 2).

Tube radius calibration. For an ideal, elastic membrane at equilibrium, the tube force F, the membrane tension σ, and the tube radius R are related as

$$R = \frac{F}{4\pi\sigma} \#(\text{S2})$$

and F and σ are measured quantities in our system. The force F in the tube can be directly measured with the optical trap and the membrane tension σ could be related with the aspiration pressure imposed by the micropipette that holds the GUV. However, this direct method is quite inexact as the absolute values of F and σ are hard to be measured with sufficient accuracy.

Alternatively, as described in Ref. (3), the radius of the nanotube can be easily obtained from the reference lipid fluorescence intensities through a previous calibration. Since the ratio of the tube lipid fluorescence $I_{\text{tube, BOD-TR}}$ and GUV lipid fluorescence $I_{\text{GUV, BOD-TR}}$ is proportional to the tube surface, and then to the tube radius, we can write the relationship

$$R = R_c \times \left(\frac{I_{\text{tube}}}{I_{\text{GUV}}}\right)_{\text{BOD-TR}} \#(\text{S3})$$

where R_c is a constant of proportionally. This constant can be determined by a linear fit of the radii values obtained from tube force and membrane tension measurements.

Bassereau and co-workers previously measured the constant R_c for GUVs containing PC lipids using the same set up. They obtained: $R_c = 200 \pm 50$ nm for DOPC with 6 independents GUVs[3], $R_c = 229 \pm 30$ nm for mixtures of EPC:EPA 9:1 with 4 independents GUVs[4], $R_c = 203 \pm 16$ nm for 57% EPC, 8% PIP2, 15% cholesterol, 10% DOPS and 10% DOPE with 11 independents GUVs[5]. All their measurements with vesicles of different formulations are consistent within a common standard deviation, typically of a 10%. For our analysis with EPC vesicles, we have used a constant value $R_c = 200 \pm 20nm$ for the calibration constant. Because the fluorescence intensities are typically measured with a higher accuracy ($\Delta I/I \approx 0.05$), the calibration formula in Eq. (S2) imposes a measurement error of $\Delta R/R = \Delta C/C \approx 0.2$ for

calculated tube radius and curvature. This measurement error is quite high and usually fixes the upper limit for the experimental accuracy in determining the curvature of the tubes.

Cardiolipin density in GUVs. In general, we define the surface density ρ as the number of molecules per area unit of membrane surface; specifically, referred to a given component j, this is $\rho_j = N_j/A$, where N_j is the number of molecules and A the total surface area. Since every molecule can be assumed to occupy an area a_j, a fractional area can be defined as $\phi_j = \rho_j a_j = A_j/A$, with $A_j = N_j a_j$ being the partial area occupied by molecules of the type-j; obviously, the total area $A = \sum_j A_j$. Referred to CL density, following the experimental procedure described in Ref. (3), a green fluorescence calibration allows us to obtain a relation between the green fluorescence intensity of the GUV and the surface density of labelled CL molecules in the membrane $\rho_{\text{GUV,Top-CL}}$. As described in Ref. (3), this can be done by using a controlled amount of a lipid dye emitting in the same channel as Top-Fluor CL. Here, we used the green fluorescent lipid BODIPY-FL HPC. We found that the measured fluorescent signal $I_{\text{GUV, BOD-FL}}$ from GUVs is linear with labelled molecule concentration up to 4500 molecules per µm^2 (see Supplementary figure 4) and then we can derive a calibration coefficient A^{gain}

$$\rho_{\text{GUV,BOD-FL}} = A^{\text{gain}} \times I^{\text{gain}}_{\text{GUV,BOD-FL}}. \#(\text{S4})$$

The proportionality constant A^{gain} depends upon both the confocal parameters (microscope objective, laser intensity, dwell time, the pinhole diameter, laser power, and PMT gain) and the BODIPY-FL HPC fluorescence efficiency. For the three gains of the confocal PMT used during experiments (100, 110, and 120) with the same excitation/acquisition conditions, we obtain: $A^{100} = 23.9 \pm 0.9$, $A^{110} = 12.6 \pm 0.7$, $A^{120} = 7.2 \pm 0.5$ (see Supplementary figure 4A). However, Eq. (S4) is only valid to quantify BODIPY-FL HPC fluorescence in the membrane. Since Top-Fluor CL has a different efficiency than BODIPY-FL HPC, the calibration has to be corrected to take into account the spectral differences of the two dyes and how these spectra are affected by the microscope optics

$$\rho_{\text{GUV,Top-CL}} = \frac{\rho_{\text{GUV,BOD-FL}}}{F^{\text{gain}}} = \frac{A^{\text{gain}} \times I^{\text{gain}}_{\text{GUV,BOD-FL}}}{F^{\text{gain}}} \#(\text{S5})$$

where $F^{\text{gain}} = I^{\text{gain}}_{\text{bulk,Top-CL}}/I^{\text{gain}}_{\text{bulk,BOD-FL}}$ quantifies the relatives fluorescence intensities of the two dyes at a given concentration. It can be deduced from the evolution of the intensity ratio measured in solution as a function of the concentration of labelled molecules in bulk. We found that both signals are linear with labelled molecule concentration (see Figures S4B-C) and then F^{gain} can be deduced from the ratio between the slopes of the linear fits $I^{\text{gain}}_{\text{bulk,Top-CL}}$ vs. $\rho_{\text{bulk,Top-CL}}$ (Supplementary figure 4C) and $I^{\text{gain}}_{\text{bulk,BOD-FL}}$ vs. $\rho_{\text{bulk,BOD-FL}}$ (Supplementary figure 4B). Finally, the area density of labelled CL in the GUV membrane, in number of molecules bound per squared micron, for the three values of PMT gains used during experiments, is

$$\rho_{\text{GUV,Top-CL}}(\mu\text{m}^{-2}) = (21.8 \pm 1.4) \times I^{100}_{\text{GUV,Top-CL}} \#(\text{S6a})$$

$$\rho_{\text{GUV,Top-CL}}(\mu\text{m}^{-2}) = (9.0 \pm 0.5) \times I^{110}_{\text{GUV,Top-CL}} \#(\text{S6b})$$ #

$$\rho_{\text{GUV,Top-CL}}(\mu\text{m}^{-2}) = (5.9 \pm 0.4) \times I^{120}_{\text{GUV,Top-CL}} \#(\text{S6c})$$

GUVs are made with 1% by mole of Top-Fluor CL and with different concentrations of CL, in particular, 0, 4%, 9%, 14%, and 24% by mole (resulting in a total CL concentration of 5%, 10%, 15%, and 25% by mole, respectively). For example, the sample with 10% of total CL contains 1% of Top-Fluor CL and 9% of CL; the sample with 25% of total CL contains 1% of Top-Fluor CL and 24% of CL. Assuming that the 1% of Top-Fluor CL represents the total CL in each sample, we can obtain an estimation of the total density of CL in the GUV membrane $\rho_{\mathrm{GUV,CL}} \equiv \rho_{\mathrm{GUV,Total\text{-}CL}}$ by multiplying the density of labelled CL by the corresponding factor 1, 5, 10, 15, or 25.

Supplementary Note 1

Experimental Data Binning

To reduce the experimental uncertainty in CL sorting data, we have binned all the raw data over four domains of tube curvatures and four domains of CL densities. Whereas the tube radius that define the curvature is more or less determined upon a fixed strength of tube pulling, the ranges of CL densities crudely correspond to the nominal compositions imposed to the lipid mixtures used to prepare the GUVs (see Supplementary table 1). We do the same for control experiment without CL (see Supplementary table 2). The bins performed to sort tube curvatures in these data sheets have been identified by their arithmetic averages (and experimental errors; second column), which are calculated over the number of GUVs contained by the specified bin (first column). The corresponding values for the CL densities $\rho_{\mathrm{GUV,CL}}$ (third column) and sorting ratio S (fourth column) are listed as statistical averages calculated from the experimental data measured by fluorescence. All the errors in these datasheets correspond to accumulated uncertainties (experimental error plus statistical variance). All these data are listed in Supplementary table 1 (for different CL contents) and Supplementary table 2 (no CL).

Supplementary Note 2

Cardiolipin Sorting Theory

There exist numerous theoretical works studying the coupling between membrane shape and composition[4,6-11]. In this section we implement a thermodynamic model for curvature-dependent CL sorting in a homogeneous binary lipid mixture EPC/CL basing on the model proposed by Aimon et al.[4]. The model determines the membrane tube composition that minimizes the total free energy of the curved membrane in which bending is modelled as a thin elastic sheet and mixing entropy is approximated by the van der Waals equation of state that includes possible CL-CL interactions. In our experiments, GUVs are made of homogeneous lipid mixtures of EPC and CL. As expected from the molecular dimensions of CL and its conical shape, the value of its intrinsic curvature should be of the order of -1 nm^{-1}, a negative value compatible with the smaller cross-sectional area of its head group relative to its hydrophobic domain[12]. Conversely, EPC lipids have a nearly cylindrical shape with an intrinsic curvature close to zero. Additionally, the area per molecule of a CL molecule is nearly a factor of two larger than that of a EPC lipid, due to its four acyl chains as compared to the two of EPC (the average cross-

sectional area of EPC[13] is around 0.69 nm^2, while it extends out almost twice for CL[14], around 1.30 nm^2). Therefore, membrane curvature should have less effect on EPC than on CL. In order to implement the model, CL molecules are assumed to insert homogeneously in both monolayers of the GUV membrane with opposite orientations due to their polar heads and their hydrophobic tails. CL molecules of the inner monolayer, which bend the membrane in the direction of the imposed curvature, are expected to be enriched in the tube while CL molecules in the outer monolayer, which bend the membrane against the imposed curvature, are expected to be depleted from the tube (see Figure 1E). Taking the Gauss convention for the extrinsic curvature of a geometrical 2D-surface embedded in R^3,[15] we denote the outer monolayer of the membrane with $(+)$, and define its mean curvature to be positive ($c^{(+)} > 0$); conversely, we denote the inner monolayer with $(-)$, as its mean curvature is negative ($c^{(-)} < 0$). The spontaneous curvature is defined in every monolayer as the result to combine the intrinsic curvature of the individual components like a set of (torsional) spring in series.[4] In general, the total spontaneous curvature of every curved monolayer (i) is related to the intrinsic curvature of the constituent molecules (c_j) as:[4]

$$c_0^{(i)} = i \sum_j \phi_j^{(i)} c_j \,, \qquad i = +, - \#(\text{S7a})$$

where the sum is referred to the j-components that compose every monolayer i being weighted by $\phi_j^{(i)} = \rho_j^{(i)} A_j^{(i)}$, which denotes the fractional area occupied by the component j in every monolayer; because $\rho_j^{(i)} = N_j^{(i)} A_j^{(i)}$consequently $\sum_j \phi_j^{(i)} = 1$. The composing molecules are characterized by an intrinsic curvature c_j, which describes its geometric aspect: $c_j > 0$ for inverted cones; $c_j = 0$ for cylinders, and $c_j < 0$ for cones. Specifically, CL is cone-shaped as far the size of its polar-head is smaller than the cross sectional area at the level of the hydrophobic tails, i.e. $c_{CL} < 0$; EPC has a cylinder aspect, so $c_{EPC} = 0$.

Therefore, when a given lipid with a non-zero intrinsic curvature inserts in a bilayer a different bending field is sensed in every monolayer; in the case considered, since $c_{CL} < 0$, the spontaneous curvature induced by CL is negative in the outer monolayer, $c_{0,CL}^{(+)} < 0$ and positive in the inner one $c_{0,CL}^{(-)} > 0$. Consequently, in the considered case, the following condition for the net curvatures $c^{(+)} - c_{0,CL}^{(+)} > c^{(-)} - c_{0,CL}^{(-)}$ necessarily holds, which imposes a prevalence for the location of CL in the inner monolayer. Because the curvature energy behaves as an harmonic field on the local curvatures, i.e. $g_{bend}^{(\pm)} = \kappa \left[c^{(\pm)} - c_{0,CL}^{(\pm)} \right]^2 / 2$, then $g_{bend}^{(+)} > g_{bend}^{(-)}$, which indicates that CL opposes to the curvature field when placed in the outer monolayer, and stabilizes the field when placed in the inner one; consequently, for CL ($c_{CL} < 0$), a spontaneous accumulation is expected in the inner monolayer followed by a depletion in the outer.

Membrane with uncoupled monolayers. In order to calculate the amount of CL molecules present in every monolayer, the total CL concentration in the membrane can be then described in terms of the surface densities $\rho_{\mathrm{CL}}^{(+)}$ and $\rho_{\mathrm{CL}}^{(-)}$. In principle, we assume that membrane monolayers are uncoupled (free to slide past each other), and then they are treated as two independent systems with their corresponding bending energies. At the end of this section (see Section S5B), we will also address an analysis when a monolayer coupling in bending energy is considered. Let's consider here the uncoupled case; if a CL molecule occupies an area A_{CL}, the

presence of a CL density $\rho_{\mathrm{CL}}^{(i)}$ in the monolayer-i lowers the EPC concentration of that monolayer by an amount

$$\frac{\rho_{\mathrm{EPC}}^{(i)}}{\rho_{\mathrm{EPC},\rho_{\mathrm{CL}}^{i}=0}^{(i)}} = 1 - A_{\mathrm{CL}}\rho_{\mathrm{CL}}^{(i)}, \qquad i = +,- \#(\mathrm{S8}) \qquad \#$$

and so, both the CL concentration and the experimentally measured sorting ratio S are mutually related by the expression

$$S = \left(\frac{\rho_{\mathrm{CL}}^{(+)} + \rho_{\mathrm{CL}}^{(-)}}{\rho_{\mathrm{EPC}}^{(+)} + \rho_{\mathrm{EPC}}^{(-)}}\right)_{\mathrm{tube}} \Bigg/ \left(\frac{\rho_{\mathrm{CL}}^{(+)} + \rho_{\mathrm{CL}}^{(-)}}{\rho_{\mathrm{EPC}}^{(+)} + \rho_{\mathrm{EPC}}^{(-)}}\right)_{\mathrm{GUV}} = \frac{\left(\rho_{\mathrm{tube,CL}}^{(+)} + \rho_{\mathrm{tube,CL}}^{(-)}\right)\left(2 - A_{\mathrm{CL}}\rho_{\mathrm{GUV,CL}}\right)}{\left[2 - A_{\mathrm{CL}}\left(\rho_{\mathrm{tube,CL}}^{(+)} + \rho_{\mathrm{tube,CL}}^{(-)}\right)\right]\rho_{\mathrm{GUV,CL}}} \#(\mathrm{S9}) \ \#$$

where we assume that $\rho_{\mathrm{GUV,CL}}^{+} = \rho_{\mathrm{GUV,CL}}^{-} = \rho_{\mathrm{GUV,CL}}/2$ (equal amount of each orientation in the GUV).

Membrane free energy. As previously noted by Aimon et al.,[4] for the experiments of lipid tube pulling it is practically sufficient to consider only the mean curvature of the membrane c. The Gaussian curvature K is zero for both the GUV (approximately flat) and the membrane tube (approximately cylindrical). Then, the membrane free energy per unit area for a curved monolayer can be written as:

$$g^{i}(c) = g_{\mathrm{mix}}^{i} + g_{\mathrm{bend}}^{i}(c), \qquad i = +,- \#(\mathrm{S10})$$

where g_{mix}^{i} is the free energy of mixing in a flat monolayer and $g_{\mathrm{bend}}^{i}(c)$ is the energy required to bend the membrane monolayer i into a cylinder with mean curvature $c^{(\pm)} = \pm 1/R$. The total free energy per unit area of the bilayer $g^{\mathrm{b}}(c)$ is the sum of the free energies of each monolayer, *i.e.*, $g^{\mathrm{b}}(c) = g^{+}\left[c^{(+)}\right] + g^{-}\left[c^{(-)}\right]$. The free energy of mixing can be approximated using the van der Waals equation of state

$$g_{\mathrm{mix}}^{i} = g_{\mathrm{ent}}^{i} + g_{\mathrm{int}}^{i} = k_B T \rho_{\mathrm{CL}}^{i} \log\left(\frac{\rho_{\mathrm{CL}}^{(i)} A_{\mathrm{CL}}}{1 - \rho_{\mathrm{CL}}^{(i)} A_{\mathrm{CL}}}\right) + a\left[\rho_{\mathrm{CL}}^{(i)}\right]^{2}, \qquad i = +,- \#(\mathrm{S11})$$

where k_B is Boltzmann's constant, T is the temperature, and a is an interaction parameter that describes the lateral interactions among CL molecules. Note we are assuming neither interaction between EPC lipids nor between EPC and CL molecules. We are also neglecting the transverse interactions between monolayers and the transbilayer (flip-flop) lipid motion (much slower process compared to sorting). The mixing energy of the bilayer $g_{\mathrm{mix}}^{\mathrm{b}}$ is the sum of the mixing energies of each monolayer, *i.e.*, $g_{\mathrm{mix}}^{\mathrm{b}} = g_{\mathrm{mix}}^{+} + g_{\mathrm{mix}}^{-}$. The values of a should impact the phase behavior of the system. In fluorescence microscopy images, the CL distribution in the GUVs appear uniform even at high CL excess (see Supplementary figure 1), therefore CL-CL interactions could only induce the formation of short-range CL clusters (below the optical limit of our confocal microscopy) possibly stabilized by long-range repulsive electrostatic interactions.

The energy required to bend the membrane into a curved shape can be estimated using the elastic response a thin, cylindrical fluid sheet. For small deflections in the harmonic regime, the monolayer bending energy per unit area is

$$g_{\text{bend}}^{i}\left[c^{(i)}\right]=\frac{\kappa^{i}}{2}\left[c^{(i)}-c_{0}^{(i)}\right]^{2}, \qquad i=+,-\#(\text{S12}) \qquad \#$$

where κ^i and $c_0^{(i)}$ are, respectively, the bending modulus and the spontaneous curvature of the monolayer i. The bending energy of the bilayer $g_{\text{bend}}^{\text{b}}(c)$ is the sum of the bending energies of each monolayer, *i.e.*, $g_{\text{bend}}^{+}\left[c^{(+)}\right]+g_{\text{bend}}^{-}\left[c^{(-)}\right]$ (recall that we are not considering any coupling between monolayers).

CL molecules can alter the bending energy by changing either the monolayer rigidity κ^i or the curvature of the monolayer in its unstressed state c_0^i. The effect of the CL molecules on the monolayer resting shape can be calculated assuming that the individual components deflect it individually as their intrinsic curvatures coupled in series (see Eq. (S7a)).[4] For the considered case, $c_{CL}<0$ and $c_{EPC}=0$, thus the total spontaneous curvature of every monolayer is related to the intrinsic curvature of CL as:

$$c_{0}^{(i)}=iA_{\text{CL}}\rho_{\text{CL}}^{i}c_{CL}, \qquad i=+,-\#(\text{S7b})$$

where c_{CL} is the intrinsic curvature of CL. Consequently, CL molecules bend the membrane in the direction of the imposed curvature ($c^{(-)}$ and c_{CL} with the same sign, inner monolayer) will drive CL enrichment while CL molecules bending the membrane against the imposed curvature ($c^{(-)}$ and c_{CL} with opposite sign, outer monolayer) will cause CL depletion.

Bending stiffness. The simplest approximation for the effective bending stiffness of a homogenous membrane composed by a binary mixture of two lipids with a different modulus was first given by Markin[16] and Helfrich and Kozlov[17] as an in-series model of mutual compliances, rationalized as the inverse rigidities of the individual components:

$$\frac{1}{\kappa^{eff}}=\frac{1-\phi}{\kappa_1}+\frac{\phi}{\kappa_2}.\#(\text{S13})$$

Here, $1-\phi$ is the area fraction occupied by the main component (1) with a bending rigidity κ_1, and ϕ represents the area fraction of the second component (2) with a bending rigidity κ_2. Eq. (S13a) predicts that κ^{eff} should follow a harmonic average of the fluid membrane and rigid inclusion moduli weighted by their respective area fractions. This ideal model has been proven to be sufficiently accurate to describe the elasticity of highly curved lipid bilayers constructed as small vesicles made of lipid binary blends,[18,19] and also used in the same context of lipid nanotubes as considered in this work.[4] In the particular case considered here, with an area fraction $\phi=A_{\text{CL}}\rho_{\text{CL}}$ occupied by CL molecules, their effect on the membrane bending modulus is given by:

$$\frac{1}{\kappa^{i}}=\frac{1-A_{\text{CL}}\rho_{\text{CL}}^{i}}{\kappa_{\text{EPC}}^{\text{m}}}+\frac{A_{\text{CL}}\rho_{\text{CL}}^{i}}{\kappa_{\text{CL}}^{\text{m}}}, \qquad i=+,-\#(\text{S14})$$

so

$$\kappa^{i}=\frac{\kappa_{\text{EPC}}^{\text{m}}\kappa_{\text{CL}}^{\text{m}}}{\kappa_{\text{CL}}^{\text{m}}+\left(\kappa_{\text{EPC}}^{\text{m}}-\kappa_{\text{CL}}^{\text{m}}\right)A_{\text{CL}}\rho_{\text{CL}}^{i}}, \qquad i=+,-\#(\text{S15}) \qquad \#$$

where the bending moduli are $\kappa_{\text{EPC}}^{\text{m}}$ for a pure EPC monolayer and $\kappa_{\text{CL}}^{\text{m}}$ for a pure CL monolayer. Because the material stiffness is considered independent of the lipid orientation, the bending

moduli $\kappa_{\mathrm{EPC}}^{\mathrm{m}}$ and $\kappa_{\mathrm{CL}}^{\mathrm{m}}$ are considered the same in both monolayers. According to Eq. (S14), in the absence of spontaneous curvature effects, the bending modulus should drive either enrichment if CL molecules soften the membrane or depletion in case of stiffening. This explains why rigid CL elicits a strong spontaneous curvature to meaningful contribute to sorting. An experimental demonstration of the increase of bending rigidity with CL content and of the quantitative validity of Eqs. (S13) and Eq. (S14) is shown in Section S6 of this Supplementary Information.

Equilibrium composition of a curved membrane. The total integrated free energy of a membrane tube with mean curvature c, surface area A_{tube} and composition $\rho_{\mathrm{tube,CL}}^{(\pm)}$, coupled to a flat GUV reservoir with area A_{GUV} and composition $\rho_{\mathrm{GUV,CL}}^{(\pm)}$, is given as:

$$G = \left\{g^{+}\left[c^{(+)}\right] + g^{-}\left[c^{(-)}\right]\right\}_{\mathrm{tube}} A_{\mathrm{tube}} + \left[g^{+}(0) + g^{-}(0)\right]_{\mathrm{GUV}} A_{\mathrm{GUV}}, \#(\mathrm{S16}) \qquad \#$$

where the curvature of the GUV is considered with a zero curvature as compared to the tube, i.e. $c_{tube}^{(\pm)} \gg c_{GUV}^{(\pm)} \approx 0$.

The integrated free energy in Eq. (S16) is referred to the total number of molecules contained in both the GUV reservoir and the tube; for every species-j contained in the monolayer i, one has $N_j^{(i)} = N_{GUV,j}^{(i)} + N_{tube,j}^{(i)} = \rho_{GUV,j}^{(i)} A_{GUV} + \rho_{tube,j}^{(i)} A_{tube}$. Assuming that mass exchanges do not occur between the two monolayers (no flip-flop), a differential transfer of molecules from the GUV reservoir to the tube would be accounted by the change $\mathrm{d}N_j^{(i)} = \mathrm{d}N_{GUV,j}^{(i)} + \mathrm{d}N_{tube,j}^{(i)} = A_{GUV}\mathrm{d}\rho_{GUV,j}^{(i)} + A_{tube}\mathrm{d}\rho_{tube,j}^{(i)}$; thus, since mass conservation imposes the condition $\mathrm{d}N_j^{(i)} = 0$ for every component, we deduce $A_{tube}\mathrm{d}\rho_{tube,j}^{(i)} = -A_{GUV}\mathrm{d}\rho_{GUV,j}^{(i)}$. Consequently, transferring a small quantity of CL molecules into a tube of fixed area A_{tube} will change the composition of both monolayers in the tube and in the GUV by the amount:

$$\mathrm{d}N_{\mathrm{CL}}^{(i)} = A_{\mathrm{GUV}}\mathrm{d}\rho_{\mathrm{GUV,CL}}^{(i)} = -A_{\mathrm{tube}}\mathrm{d}\rho_{\mathrm{tube,CL}}^{(i)} \qquad i = +,- \#(\mathrm{S17}) \qquad \#$$

Therefore, the corresponding change in free energy is:

$$\begin{aligned}
\mathrm{d}G &= \left[\frac{\partial g^{+}\left[c^{(+)}\right]}{\partial \rho_{\mathrm{CL}}^{(+)}}\mathrm{d}\rho_{\mathrm{CL}}^{(+)} + \frac{\partial g^{-}\left[c^{(-)}\right]}{\partial \rho_{\mathrm{CL}}^{(-)}}\mathrm{d}\rho_{\mathrm{CL}}^{(-)}\right]_{\mathrm{tube}} A_{\mathrm{tube}} \\
&\quad + \left[\frac{\partial g^{+}(0)}{\partial \rho_{\mathrm{CL}}^{(+)}}\mathrm{d}\rho_{\mathrm{CL}}^{(+)} + \frac{\partial g^{-}(0)}{\partial \rho_{\mathrm{CL}}^{(-)}}\mathrm{d}\rho_{\mathrm{CL}}^{(-)}\right]_{\mathrm{GUV}} A_{\mathrm{GUV}} \\
&= \left[\frac{\partial g_{\mathrm{tube}}^{(+)}\left[c^{(+)}\right]}{\partial \rho_{\mathrm{tube,CL}}^{(+)}} - \frac{\partial g_{\mathrm{GUV}}^{(+)}(0)}{\partial \rho_{\mathrm{GUV,CL}}^{(+)}}\right]\mathrm{d}N_{\mathrm{CL}}^{(+)} + \left[\frac{\partial g_{\mathrm{tube}}^{(-)}\left[c^{(-)}\right]}{\partial \rho_{\mathrm{tube,CL}}^{(-)}} - \frac{\partial g_{\mathrm{GUV}}^{(-)}(0)}{\partial \rho_{\mathrm{GUV,CL}}^{(-)}}\right]\mathrm{d}N_{\mathrm{CL}}^{(-)}
\end{aligned}$$

$$\#(\mathrm{S18})$$

where

$$\frac{\partial g^{i}\left[c^{(i)}\right]}{\partial \rho_{\mathrm{CL}}^{(i)}} = -\kappa^{i}\left[c^{(i)} - c_0^{(i)}\right] A_{\mathrm{CL}} c_{\mathrm{CL}}^{(i)} + \frac{\left(\kappa^{i}\right)^2\left[c^{(i)} - c_0^{(i)}\right]^2}{2\kappa_{\mathrm{EPC}}^{\mathrm{m}}}\left(1 - \frac{\kappa_{\mathrm{EPC}}^{\mathrm{m}}}{\kappa_{\mathrm{CL}}^{\mathrm{m}}}\right) A_{\mathrm{CL}}$$

$$+k_BT\left[\log\left(\frac{\rho_{\mathrm{CL}}^{(i)}A_{\mathrm{ref}}}{1-\rho_{\mathrm{CL}}^{i}A_{\mathrm{CL}}}\right)+1+\frac{\rho_{\mathrm{CL}}^{(i)}A_{\mathrm{CL}}}{1-\rho_{\mathrm{CL}}^{i}A_{CL}}\right]+a\rho_{\mathrm{CL}}^{(i)}, \qquad i=+,- \tag{S19}$$

where κ^i is given by Eq. (S14).

At equilibrium, the chemical potentials equal between the tube and the GUV, so:

$$\left[\frac{\partial g[c^{(i)}]}{\partial \rho_{\mathrm{CL}}}\right]_{tube}=\left[\frac{\partial g(0)}{\partial \rho_{\mathrm{CL}}}\right]_{GUV}, \qquad i=+,- \tag{S20}$$

Finally, we have to solve the Eq. (S19) for both monolayers ($+$ and $-$) to obtain $\rho_{\mathrm{tube,CL}}^{+}$ and $\rho_{\mathrm{tube,CL}}^{-}$, and then replace them in Eq. (S8) to determine sorting. There is no simple analytical solution for this equation but numerical solutions can be obtained. Due to the difference by three orders of magnitude in area between the GUV and the tube, the change in GUV composition can be neglected and can be considered as a chemostat (reservoir) for the tube. The, the CL densities in the GUV can be assumed to remain approximately constant and equal to $\rho_{\mathrm{GUV,CL}}^{+}=\rho_{\mathrm{GUV,CL}}^{-}=\rho_{\mathrm{GUV,CL}}/2$ (equal amount of each orientation).

Estimation of the monolayer bending modulus. The uncoupled model requires to know the bending moduli of EPC and CL monolayers. However, the easily measured quantity is the bilayer bending modulus, but not so much the monolayer one, and no data are available about the bending modulus of pure CL monolayers. In order to estimate these parameters, we write the bending energy of the bilayer $g_{\mathrm{bend}}^{\mathrm{b}}$ as the sum of the bending energies of the monolayers $+$ and $-$ (uncoupled case)

$$g_{\mathrm{bend}}^{\mathrm{b}}=\frac{1}{2}\kappa^{\mathrm{b}}\left(c-c_0^{\mathrm{b}}\right)^2=g_{\mathrm{bend}}^{+}+g_{\mathrm{bend}}^{-}=\frac{1}{2}\kappa^{+}\left[c^{(+)}-c_0^{(+)}\right]^2+\frac{1}{2}\kappa^{-}\left[c^{(-)}-c_0^{(-)}\right]^2 \tag{S21}$$

where κ^{b} is the bilayer bending modulus, c the mean curvature of the bilayer and c_0^{b} its spontaneous curvature, which corresponds to its unstressed, resting shape.

For a bilayer in which CL inserts homogeneously with both orientations in the two monolayers, we have $\kappa^{\mathrm{m}}\equiv\kappa^{+}=\kappa^{-}$, $c^{m}\equiv c^{(+)}=-c^{(-)}$ and $c_0^{\mathrm{m}}\equiv c_0^{(+)}=-c_0^{(-)}$. Therefore, according to Eq. (S21), $c_0^{\mathrm{b}}=0$ by taking Eq. (S20), then

$$g_{\mathrm{bend}}^{\mathrm{b}}=\frac{1}{2}\kappa^{\mathrm{b}}c^2=\kappa^{\mathrm{m}}[c^2+(c_0^{\mathrm{m}})]=\kappa^{\mathrm{m}}c^2+\text{constant}, \tag{S22}$$

where the constant term just represents a shift in the origin of free energy. Consequently, Eq. (S22) shows as the bending modulus of an uncoupled monolayer can be approximated by half the bending modulus of a bilayer with the same composition, this is:

$$\kappa^{\mathrm{m}}=\kappa^{\mathrm{b}}/2\,. \tag{S23}$$

If the monolayers are somewhat coupled, Eq. (S23) does not hold since the bending energy cannot be written as the sum of the bending energies of the monolayers [Eq. (S20)].

Experimental values from literature. Implementation of the theoretical model requires to introduce the following parameters: A_{CL}, area of a CL molecule; $\kappa_{\mathrm{EPC}}^{\mathrm{m}}$, bending modulus of a

pure EPC monolayer; $\kappa_{\rm CL}^{\rm m}$, bending modulus of a pure CL monolayer; $c_{\rm CL}$, intrinsic curvature of CL; and a, the CL-CL interaction term. Except for the interaction term, there exist previous experimental measurements of these parameters. On one side, scattering analysis gave a CL area[14] of approximately 1.3 nm^2, which is nearly a factor of two larger than that of a EPC lipid[13] (around 0.69 nm^2), due to its four acyl chains as compared to the two of EPC. On other side, flickering spectroscopy measurements of CL bilayers yield a bending modulus[14] $\kappa_{\rm CL}^{\rm b} = 1.06 \times 10^{-19}$ J $\approx 26\, k_B T$ at $T = 298$ K, which is larger than that observed in EPC bilayers[15] $\kappa_{\rm EPC}^{\rm b} = 10 \pm 1\, k_B T$ at $T = 298$ K. According to Eq. (S22), the bending modulus of an uncoupled monolayer can be estimated as $\kappa^{\rm m} = \kappa^{\rm b}/2$, and then $\kappa_{\rm EPC}^{\rm m} = 5\, k_B T$ and $\kappa_{\rm CL}^{\rm m} = 13\, k_B T$ at $T = 298\, K$. The fit to the observed data using the previous experimental values will provide the expected CL curvature and the range of CL clusters. The CL intrinsic curvature obtained from the fit can be then compared with previous estimations[12], which suggest that it should be of the order of 1 nm^{-1} due to the smaller cross-sectional area of its head group relative to its hydrophobic domain.

Sorting as a function of tube curvature. Figure 2 shows the CL enrichment as a function of the area fraction $\rho_{\rm GUV,CL} \times A_{\rm CL}$ (which corresponds to the fraction of the GUV surface covered with CL molecules) for four ranges of tube curvature (see Supplementary table 1). The minimum square fit to the observed data in the absence of binary interactions between CL molecules (*i.e.*, interacting parameter $a = 0$) gives $c_{\rm CL} = -1.12 \pm 0.4$ nm^{-1} (dashed lines) and assuming possible CL-CL interactions gives $c_{\rm CL} = -1.10 \pm 0.05$ nm^{-1} and $a = (-18 \pm 1)\, k_B T$ nm^2 (solid lines). Clearly, the non-interacting uncoupled model fails to reproduce the experimental data, for any values of the tube curvature (c). Although the non-interacting uncoupled model describes a curvature-increasing sorting at low CL-content, it largely underestimates the results at intermediate and high CL concentration. In contrast, interacting uncoupled model is successful in reproducing the experimental measurements. This conclusion can be also extracted from Supplementary figure 5, where it is represented the CL enrichment as a function of the tube curvature for four ranges of the area fraction.

Tube spontaneous curvature and bending modulus. The flat membrane of the GUV is assumed to contain CL molecules at equal density in every monolayer, $\rho_{\rm CL,GUV}^{+} = \rho_{\rm CL,GUV}^{-}$. Since the GUV can be considered a reservoir for the tube, its CL densities can be assumed to remain approximately constant and equal, this is $\rho_{\rm GUV,CL}^{+} = \rho_{\rm GUV,CL}^{-} = \rho_{\rm GUV,CL}/2$ (equal amount of each orientation). As a consequence, the spontaneous curvature of the GUV is zero [Eq. (S12)] and the bending modulus of the GUV is equal in both monolayers and depends on $\rho_{\rm GUV,CL}$ according to Eq. (S14). However, in the highly curved tube, because the negative value of the intrinsic curvature ($c_{\rm CL} = -1.10$ nm^{-1}), the model predicts CL molecules at a higher density in the inner monolayer than in the outer one (see Figure 3). These opposite effects of curvature on the two monolayers do not cancel each other and so the total CL concentration of the bilayer increases with curvature. Because $c_{\rm CL} < 0$, the spontaneous curvature in the tube [Eq. (S12)] is positive in the inner monolayer and negative in the outer one (Supplementary figure 6A), but its absolute value varies differently in every monolayer as CL concentrates at the inner one and becomes depleted from the outer one. Additionally, CL globally stiffens the membrane, thus the effective value of the bending modulus of the tube [Eq. (S15)] increases with curvature as the density of CL increases in the inner monolayer and decreases in the outer one (Supplementary figure 6B).

Analysis of the bending, entropic, and interaction contributions to sorting. In this section, we

address a deeper analysis of the bending, entropic, and interaction contributions to sorting and study how they are affected by CL density. To do that, we recall the expression of the chemical potential $\mu^i[c^{(i)}] \equiv \partial g^i[c^{(i)}]/\partial\rho^i$, defined as the rate of change of the total free energy of a system with respect to the change in the number of molecules that are added to it. From Eq. (S18) we have

$$\mu^i[c^{(i)}] \equiv \frac{\partial g^i[c^{(i)}]}{\partial\rho^i_{\mathrm{CL}}} = -\kappa^i\left(c^{(i)} - c_0^{(i)}\right)A_{\mathrm{CL}}c_{\mathrm{CL}}^{(i)} + \frac{\left(\kappa^i\right)^2\left(c^{(i)} - c_0^i\right)^2}{2\kappa^{\mathrm{m}}_{\mathrm{EPC}}}\left(1 - \frac{\kappa^{\mathrm{m}}_{\mathrm{EPC}}}{\kappa^{\mathrm{m}}_{\mathrm{CL}}}\right)A_{\mathrm{CL}}$$
$$+k_BT\left[\log\left(\frac{\rho^i_{\mathrm{CL}}A_{\mathrm{ref}}}{1-\rho^i_{\mathrm{CL}}A_{\mathrm{CL}}}\right) + 1 + \frac{\rho^i_{\mathrm{CL}}A_{\mathrm{CL}}}{1-\rho^i_{\mathrm{CL}}A_{\mathrm{CL}}}\right] + a\rho^i_{\mathrm{CL}}, \qquad i = +,- \#(\mathrm{S24})$$

where the bending, entropic and interaction terms are written in blue, red, and green, respectively. The equilibrium condition in our system is given by [Eq. (S19)]

$$0 = \Delta\mu^i = \mu^i_{\mathrm{tube}} - \mu^i_{\mathrm{GUV}}$$
$$= \left[\kappa^i_{\mathrm{tube}}\left(c^{(i)} - c^{(i)}_{0,\mathrm{tube}}\right) - \kappa^i_{\mathrm{GUV}}c^{(i)}_{0,\mathrm{GUV}}\right]A_{\mathrm{CL}}C^i_{\mathrm{CL}} + \left[\frac{\left(\kappa^i\right)^2\left(c^{(i)} - c^{(i)}_{0,\mathrm{tube}}\right)^2}{2\kappa^{\mathrm{m}}_{\mathrm{EPC}}}\right.$$
$$\left.-\frac{\left(\kappa^i_{\mathrm{GUV}}\right)^2\left(c^{(i)}_{0,\mathrm{GUV}}\right)^2}{2\kappa^{\mathrm{m}}_{\mathrm{EPC}}}\right]\left(1 - \frac{\kappa^{\mathrm{m}}_{\mathrm{EPC}}}{\kappa^{\mathrm{m}}_{\mathrm{CL}}}\right)A_{\mathrm{CL}} + k_BT\left\{\log\left[\frac{\rho^i_{\mathrm{tube,CL}}\left(1-\rho^i_{\mathrm{GUV,CL}}A_{\mathrm{CL}}\right)}{\left(1-\rho^i_{\mathrm{tube,CL}}A_{\mathrm{CL}}\right)\rho^i_{\mathrm{GUV,CL}}}\right]\right.$$
$$\left.+\frac{\rho^i_{\mathrm{tube,CL}}A_{\mathrm{CL}}}{1-\rho^i_{\mathrm{tube,CL}}A_{\mathrm{CL}}} - \frac{\rho^i_{\mathrm{GUV,CL}}A_{\mathrm{CL}}}{1-\rho^i_{\mathrm{GUV,CL}}A_{\mathrm{CL}}}\right\} + a\left(\rho^i_{\mathrm{tube,CL}} - \rho^i_{\mathrm{GUV,CL}}\right), \qquad i = +,- \#(\mathrm{S25})$$

A positive difference in Eq. (S25) ($\Delta\mu^i > 0$) implies a net flux of CL molecules from the tube to the GUV, decreasing sorting, while a negative difference ($\Delta\mu^i < 0$) implies a net flux of CL molecules from the GUV to the tube, increasing sorting. As we can see in Figure 1E, CL molecules in the inner monolayer $i = +$, which bend the membrane in the direction of the imposed curvature, are enriched in the tube, while CL molecules in the outer monolayer $i = -$, which bend the membrane against the imposed curvature, are depleted from the tube. Then, we can write

$\Delta\mu^- > 0$ (outer monolayer) → net flux of CL from the tube to the GUV → sorting decrease#(S26a)
$\Delta\mu^+ < 0$ (inner monolayer) → net flux of CL from the GUV to the tube → sorting increase#(S26b)

Once the system is at equilibrium the net flux of both monolayers is zero. We can determine how bending, entropic, and interactive terms alter sorting by analyzing the sign of their corresponding chemical potential differences. Since the CL density in the outer monolayer of the tube $\rho^-_{\mathrm{tube,CL}}$ is much lower than that in inner monolayer $\rho^+_{\mathrm{tube,CL}}$ (see Figure 3), it is sufficient to evaluate these terms in the inner monolayer Eq. (S26b). Thus, we have to calculate the following expressions

$$\Delta\mu^+_{\mathrm{bend}} = \Delta\mu^+_{\mathrm{bend},c_0} + \Delta\mu^+_{\mathrm{bend},\kappa} = -\left[\kappa^+_{\mathrm{tube}}\left(c^{(i)} - c^{(+)}_{0,\mathrm{tube}}\right) + \kappa^+_{\mathrm{GUV}}c^{(+)}_{0,\mathrm{GUV}}\right]A_{\mathrm{CL}}c^{(+)}_{\mathrm{CL}}$$
$$+\frac{\left(\kappa^+_{\mathrm{tube}}\right)^2\left(c^{(i)} - c^{(+)}_{0,\mathrm{tube}}\right)^2 - \left(\kappa^+_{\mathrm{GUV}}\right)^2\left(c^{(+)}_{0,\mathrm{GUV}}\right)^2}{2\kappa^{\mathrm{m}}_{\mathrm{EPC}}}\left(1 - \frac{\kappa^{\mathrm{m}}_{\mathrm{EPC}}}{\kappa^{\mathrm{m}}_{\mathrm{CL}}}\right)A_{\mathrm{CL}}, \#(\mathrm{S27})$$

$$\Delta\mu_{\mathrm{ent}}^{+} = k_BT\left[\log\left[\frac{\rho_{\mathrm{tube,CL}}^{+}\left(1-\rho_{\mathrm{GUV,CL}}^{+}A_{\mathrm{CL}}\right)}{\left(1-\rho_{\mathrm{tube,CL}}^{+}A_{\mathrm{CL}}\right)\rho_{\mathrm{GUV,CL}}^{+}}\right]+\frac{\rho_{\mathrm{tube,CL}}^{+}A_{\mathrm{CL}}}{1-\rho_{\mathrm{tube,CL}}^{+}A_{\mathrm{CL}}}-\frac{\rho_{\mathrm{tube,CL}}^{+}A_{\mathrm{CL}}}{1-\rho_{\mathrm{GUV,CL}}^{+}A_{\mathrm{CL}}}\right], \#(\mathrm{S28})$$

$$\Delta\mu_{\mathrm{int}}^{+} = a\left(\rho_{\mathrm{tube,CL}}^{+}-\rho_{\mathrm{GUV,CL}}^{+}\right), \#(\mathrm{S29})$$

where the bending contribution to sorting has been separated into the spontaneous curvature contribution (written above in purple)

$$\Delta\mu_{\mathrm{bend},c_0}^{+} = \frac{\partial g_{\mathrm{bend}}^{+}}{\partial c_0^{(+)}}\frac{\partial c_0^{(+)}}{\partial \rho_{\mathrm{CL}}^{+}}, \#(\mathrm{S30})$$

and the bending modulus contribution (written above in cyan)

$$\Delta\mu_{\mathrm{bend},\kappa}^{+} = \frac{\partial g_{\mathrm{bend}}^{+}}{\partial \kappa^{+}}\frac{\partial \kappa^{+}}{\partial \rho_{\mathrm{CL}}^{+}}. \#(\mathrm{S31})$$

The chemical potential differences of these individual contributions as a function of CL density for a tube curvature of 0.1 nm^{-1} are shown in Supplementary figure 7. Curves fitting to the interacting uncouple model ($c_{\mathrm{CL}} = -1.10\ \mathrm{nm}^{-1}$ and $a = -18\ k_BT\ \mathrm{nm}^2$) are represented with solid lines while curves fitting to the non-interacting uncoupled model ($c_{\mathrm{CL}} = -1.12\ \mathrm{nm}^{-1}$ and $a = 0$) are represented with dashed lines. In both cases sorting is limited by the entropic term since $\Delta\mu_{\mathrm{ent}}^{+} > 0$ (red lines in Supplementary figure 7A). This is something expected since mixing entropy tends to homogenize the lipid distribution. The bending contribution to sorting is, however, quite different between the interacting and the non-interacting uncoupled models: when non-CL interaction is assumed, the bending term (blue dashed line in Supplementary figure 7A) contributes slightly to sorting ($\Delta\mu_{\mathrm{bend}}^{+} \lessapprox 0$) and this contribution diminishes as CL density increases. In contrast, when CL-CL attractive interactions are considered, the bending term enhances CL enrichment just for very low CL densities (up to the product $\rho_{\mathrm{GUV,CL}} \times A_{\mathrm{CL}}$ is approximately 0.1 in Supplementary figure 7A). Above this value, the bending term drives CL depletion. High CL accumulation in the tube minimizes its curvature frustration but increases its bending modulus ($\kappa_{\mathrm{CL}} > \kappa_{\mathrm{EPC}}$). The resulting CL enrichment is then enhanced by the spontaneous curvature term ($\Delta\mu_{\mathrm{bend},c_0}^{+} < 0$, purple line in Supplementary figure 7B) but limited by the stiffness penalty ($\Delta\mu_{\mathrm{bend},\kappa}^{+} > 0$, cyan line in Supplementary figure 7B). The interaction term (green solid line in Supplementary figure 7A) contributes to a strong CL enrichment ($\Delta\mu_{\mathrm{int}}^{+} < 0$) and then it is required to explain the observed data at high densities. Finally, $\Delta\mu_{\mathrm{total}}^{+} = 0$ (black line) since the calculations are made when the system is at equilibrium.

Additionally, Supplementary figure 8 shows the influence of the bending, entropic and interacting contributions to sorting as a function of tube curvature for very low (Supplementary figure 8A) and very high (Supplementary figure 8B) CL densities. At low CL densities, the influence of CL interaction (green solid line) is negligible, thereby there are no significant differences between the results of the non-interacting (dashed lines) and the interacting (solid lines) uncoupled models. Entropy drives CL depletion ($\Delta\mu_{\mathrm{ent}}^{+} > 0$) and bending contributes slightly to sorting ($\Delta\mu_{\mathrm{bend}}^{+} < 0$). In contrast, at high densities, there exist quite differences between the interacting and the non-interacting uncoupled models and CL-CL attractive interactions are essential to explain the observed data.

Membrane with coupled monolayers. Previous sections analyze the sorting model when both

membrane monolayers are assumed to be uncoupled and therefore the bending energy of the bilayer can be written as the sum of the bending energies of the monolayers [Eq. (S20)]. In order to get insight about the coupled/uncoupled behavior of the membrane, we have also performed the fit to a membrane model with monolayer coupling in the bending energy, specifically, in the bending modulus. In this case, the bending energy of the bilayer is

$$g_{\mathrm{bend}}^{\mathrm{b}}(c) = \frac{1}{2}\kappa^{\mathrm{b}}\left(c - c_0^{\mathrm{b}}\right)^2. \#(\mathrm{S32})$$

As in the uncoupled case, the bilayer spontaneous curvature is, in a simple approach, proportional to the differences in the CL content in the two monolayers[20]

$$c_0^{\mathrm{b}} = c_0^{(+)} + c_0^{(-)} = A_{\mathrm{CL}}\rho_{\mathrm{CL}}^{+}c_{CL} - A_{\mathrm{CL}}\rho_{\mathrm{CL}}^{-}c_{CL} = A_{\mathrm{CL}}c_{\mathrm{CL}}\left(\rho_{\mathrm{CL}}^{+} - \rho_{\mathrm{CL}}^{-}\right). \#(\mathrm{S33})$$

However, unlike the uncoupled case, the bending modulus of a coupled membrane can be obtained as

$$\kappa^{\mathrm{b}} = \frac{\kappa_{\mathrm{EPC}}^{\mathrm{b}}}{1 - \left(1 - \kappa_{\mathrm{EPC}}^{\mathrm{b}}/\kappa_{\mathrm{CL}}^{\mathrm{b}}\right)A_{\mathrm{CL}}\left(\rho_{\mathrm{CL}}^{+} + \rho_{\mathrm{CL}}^{-}\right)}. \#(\mathrm{S34})$$

The mixing energy of the bilayer [Eq. (S10)] is the same in both uncoupled and coupled model since we are neglecting the transbilayer CL motion and the interaction between monolayers. In other words, we are considering that CL molecules can only mix and interact within their monolayer. The rest of equations written in Section 5A for the uncoupled model are also valid here replacing the monolayer spontaneous curvature [Eq. (S12)] and the monolayer bending modulus [Eq. (S14)] by the bilayer ones [Eqs. (S33) and (S34), respectively]. Now, instead of having two independent equations [Eq. (S19)] ($i = +$ which gives $\rho_{\mathrm{tube,CL}}^{+}$ and $i = -$ which gives $\rho_{\mathrm{tube,CL}}^{-}$) we have two coupled equations with two parameters $\rho_{\mathrm{tube,CL}}^{+}$ and $\rho_{\mathrm{tube,CL}}^{-}$.

Although the coupled model also predicts high sorting at high CL densities when we assume CL-CL interactions (solid lines of Supplementary figure 9), it does not reproduce our experimental data at high curvatures (brown solid line of Supplementary figure 9). The minimum square fit to the coupled model in the absence of binary interactions between CL molecules (*i.e.*, interacting parameter $a = 0$) is represented in Supplementary figure 9 with dashed lines and gives an intrinsic curvature of CL of $c_{\mathrm{CL}} = -0.7 \pm 0.2\ \mathrm{nm}^{-1}$. The minimum square fit to the coupled model possible CL-CL interactions is represented in Supplementary figure 9 with solid lines and gives $c_{\mathrm{CL}} = -0.6 \pm 0.1\ \mathrm{nm}^{-1}$ and $a = (-9.4 \pm 0.4)\ k_B T\ \mathrm{nm}^2$. By comparing the uncoupled (Figure 2) and coupled results, it appears that the EPC/CL bilayers do not have significant coupling in bending modulus, thereby both monolayers can be treated as independent systems, like occurs in other lipid bilayers in the absence of significantly flip-flopping lipids.[21]

Supplementary Discussion

Mechanical parameters of the CL+EPC binary system

Encouraged by the need to verify the likelihood of the mechanical model underlying Eqs. (S13)-(S15), and to give experimental support to the hypothesis of a weak intermonolayer coupling leading to a best fit of the experimental results to the uncoupled monolayer model, we studied

the mechanical impact of including CL in lipid membranes based on EPC. Two model systems were constructed: i) Bilayer vesicles to measure bending rigidities via shape fluctuations (see Section S6A).[22,23] ii) Langmuir monolayers to measure the compression modulus, and so analyze its quantitative relationship with the bilayer bending modulus through the degree of coupling between the two monolayers (see Section S6B).[24]

Bending modulus of the CL-modified bilayers. We prepared giant unilamellar vesicles (GUVs) made of binary mixtures of EPC and CL in the whole range of molar compositions (see Methods). Then, we take advantage of the fluctuation spectroscopy (FS) method to measure the bending modulus (κ) of the mixed lipid bilayers prepared with variable amounts of CL (extensive account of the FS method is given in Ref. (23)). The vesicles made of the bare EPC were characterized by a relatively low value of the bending modulus, $\kappa_{EPC} = 10 \pm 4\, k_B T$ ($N = 15$ vesicles), in agreement with literature results.[13,14] The vesicles made of bare CL, although mechanically more unstable due to its quasi-solid character, were characterized by a higher bending stiffness, $\kappa_{CL} = 31 \pm 15\, k_B T$ ($N = 6$ vesicles). This value is also in quantitative agreement with previous data from structural experiments.[14] Both results support the reliability of the specific values of κ_{CL} and κ_{EPC} considered in our models. Supplementary figure 10A shows the experimental values of $\kappa(\phi)$ measured for the vesicles made of the binary mixtures as a function of the CL area fraction (ϕ). A monotonic increase between the extremal values is observed in a dependence that is compatible with the linear compliance model described by Eq. S13, which has been used to evaluate the dependence of the bending modulus of the monolayers on the CL content. Although the results above support themselves the validity of the material model used to describe the bending rigidity in our theory, an additional evidence on the degree of coupling between the monolayers would be highly acknowledged

Mechanical parameters of the CL-modified membranes. We used the Langmuir trough to form insoluble monolayers of the lipid blend for getting the pressure-area isotherm ($\pi - A$ curve) measured upon lateral compression.[24] This configuration is optimal to measure the lateral compression modulus of the lipid monolayer (K_m), which is defined as:

$$K_m = -\frac{1}{A}\left(\frac{\partial \pi}{\partial A}\right)_T \tag{S35}$$

Supplementary figure 10B shows the correlation between the experimental values of the bending rigidity (from Fig. S10A) and those of the compression modulus measured with the Langmuir balance at a surface pressure compatible with the bilayer assembly ($\pi_b = 30\, mN/m$). The mechanical interplay between the bending stiffness of a lipid bilayer and the compression rigidity of the individual monolayers is mediated by normal stresses that held them together giving rise to intermonolayer coupling. Depending on the degree of intermonolayer coupling, the monolayers are more or less restrained relative each other. This coupling results in a linear relation between the bending modulus (κ) and the compression modulus of the monolayers (K_m), which is dependent on the bilayer thickness h as:[25]

$$\kappa(h) = \frac{(h-h_0)^2}{\alpha} K_m \tag{S36}$$

where the difference $h - h_0$ accounts for the effective hydrophobic thickness, and α is a coupling constant, which takes values between two extreme cases: i) Ideally coupled bilayer model ($\alpha \leq 12$), corresponding to full coupling between the two monolayers; ii) uncoupled model ($\alpha \geq 48$), corresponding to two monolayers able to freely slide past each other. The brush-like model accounts for intermediate coupling scenarios[26], where the two monolayers couple at a variable strength depending on their degree of mutual interaction.

Although all the results in Fig. S10B lye in the intermediate coupling region accounted for by the brush-like model, the membranes based on EPC with a low CL content ($\phi \leq 40\%$) are characterized by a relatively low value of the coupling constant compatible with the uncoupled monolayer model. With regard to the conclusions raised in our work, this experimental evidence confirms the assumed hypothesis of a weak intermonolayer coupling do not favoring material exchange between the monolayers.

Supplementary References

(1) Angelova MI, Soléau S, Méléard P, Faucon F, Bothorel P (1992) Preparation of giant vesicles by external AC electric fields. Kinetics and applications in Trends in Colloid and Interface Science VI. (Steinkopff, Darmstadt), pp. 127–131.

(2) Prévost C, Tsai FC, Bassereau P, Simunovic M (2017) Pulling Membrane Nanotubes from Giant Unilamellar Vesicles. Journal of Visualized Experiments (130):e56086.

(3) Sorre B, et al. (2012) Nature of curvature coupling of amphiphysin with membranes depends on its bound density. Proceedings of the National Academy of Sciences 109(1):173–178.

(4) Aimon S, et al. (2014) Membrane Shape Modulates Transmembrane Protein Distribution. Developmental Cell 28(2):212–218.

(5) Prévost C, et al. (2015) IRSp53 senses negative membrane curvature and phase separates along membrane tubules. Nature Communications 6(1):8529.

(6) Cooke IR, Deserno M (2006) Coupling between lipid shape and membrane curvature. Biophysical journal 91(2):487–495.

(7) Derganc J (2007) Curvature-driven lateral segregation of membrane constituents in Golgi cisternae. Physical Biology 4(4):317–324.

(8) Leibler S (1986) Curvature instability in membranes. Journal de Physique 47(3):507–516.

(9) Markin V (1981) Lateral organization of membranes and cell shapes. Biophysical Journal 36(1):1–19.

(10) Seifert U (1993) Curvature-induced lateral phase segregation in two-component vesicles. Physical Review Letters 70(9):1335–1338.

(11) Singh P, Mahata P, Baumgart T, Das SL (2012) Curvature sorting of proteins on a cylindrical lipid membrane tether connected to a reservoir. Physical Review E 85(5):051906.

(12) Renner LD, Weibel DB (2011) Cardiolipin microdomains localize to negatively curved regions of Escherichia coli membranes. Proceedings of the National Academy of Sciences 108(15):6264–6269.

(13) Kucerka N, Tristram-Nagle S, Nagle JF (2005) Structure of Fully Hydrated Fluid Phase Lipid Bilayers with Monounsaturated Chains. J. Membrane Biol. 208(3):193–202.

(14) Pan J, et al. (2015) Structural and mechanical properties of cardiolipin lipid bilayers determined using neutron spin echo, small angle neutron and X-ray scattering, and molecular dynamics simulations. Soft Matter 11(1):130–138.

(15) Do Carmo M. Diﬀerential Geometry of Curves and Surfaces (Prentice-Hall, Englewood Cliﬀs, 1976)

(16) Markin VS (1981) Lateral organization of membranes and cell shapes. Biophys. J. 36:1–19.

(17) Helfrich W and MM Kozlov (1994) Flexibility and roughness of mixed and partially polymerized bilayers in terms of the hat model and local bending frustration. J. Phys. II 4:1427–38.

(18) Arriaga LR et al. (2009) Stiffening effect of cholesterol on disordered lipid phases: a combined neutron spin echo+ dynamic light scattering analysis of the bending elasticity of large unilamellar vesicles. Biophys J 96(9):3629-37

(19) Kelley EG, PD Butler and M. Nagao (2019) Scaling of lipid membrane rigidity with domain area fraction. Soft Matter 15:2762-67

(20) Sorre B, et al. (2009) Curvature-driven lipid sorting needs proximity to a demixing point and is aided by proteins. Proceedings of the National Academy of Sciences of the United States of America 106(14):5622–5626.

(21) Arriaga LR, et al. (2017) Dissipative dynamics of fluid lipid membranes enriched in cholesterol. Advances in Colloid and Interface Science 247:514–520.

(22) Rodríguez-García R et al. (2011) Subdiffusive fluctuation dynamics of rigid membranes as resolved by ultrafast videomicroscopy. EPL (Europhysics Letters) 94(2): 28009

(23) Mell M and F Monroy (2018) A gradient-based, GPU-accelerated, high-precision contour-segmentation algorithm with application to cell membrane fluctuation spectroscopy. PloS One 13 (12): e0207376

(24) López-Montero I, et al (2010) Lipid domains and mechanical plasticity of Escherichia coli lipid monolayers. Chem. Phys. Lipids 163(1):56-63

(25) Helfrich W (1973) Elastic properties of lipid bilayers: Theory and possible experiments Naturforsch. C 28: 693-703

(24) Rawicz W et al. (2000) Effect of chain length and unsaturation on elasticity of lipid bilayers. Biophys J. 79:328–39
